# Bichloride-based ionic liquids for the merged storage, processing, and electrolysis of hydrogen chloride

**DOI:** 10.1126/sciadv.adn5353

**Published:** 2024-04-03

**Authors:** Gesa H. Dreyhsig, Patrick Voßnacker, Merlin Kleoff, Haralds Baunis, Niklas Limberg, Michael Lu, Reinhard Schomäcker, Sebastian Riedel

**Affiliations:** ^1^ Freie Universität Berlin, Institut für Anorganische Chemie, Fabeckstr. 34/36, 14195 Berlin, Germany.; ^2^ Technische Universität Berlin, Institut für Technische Chemie, Straße des 17. Juni 124, 10623 Berlin, Germany.

## Abstract

Hydrogen chloride is produced as a by-product in industrial processes on a million-ton scale. Since HCl is inherently dangerous, its storage and transport are avoided by, e.g., on-site electrolysis providing H_2_ and Cl_2_ which usually requires complex cell designs and PFAS-based membranes. Here we report a complementary approach to safely store 0.61 kilogram HCl per kilogram storage material [NEt_3_Me]Cl forming the bichloride [NEt_3_Me][Cl(HCl)*_n_*]. Although HCl release is possible from this ionic liquid by heat or vacuum, the bichloride can be used directly to produce base chemicals like vinyl chloride. Alternatively, [NEt_3_Me][Cl(HCl)*_n_*] is electrolyzed under anhydrous conditions using a membrane-free cell to generate H_2_ and the corresponding chlorination agent [NEt_3_Me][Cl(Cl_2_)*_n_*], enabling the combination of these ionic liquids for the production of base chemicals.

## Teaser

Ionic liquids complement the established systems of gaseous and aqueous HCl.

## INTRODUCTION

Chlorine is one of the most important base chemicals (~100 million t a^−1^ worldwide) and is required for the production of intermediate chemicals, e.g., chloromethanes and for indispensable polymers such as polyurethanes (PU) and polycarbonates (*1*–*6*). During the production of only these three materials, in total, 9.3 million t a^−1^ of hydrogen chloride (HCl) are released as a by-product (*5*–*7*).

**Fig. 1.**
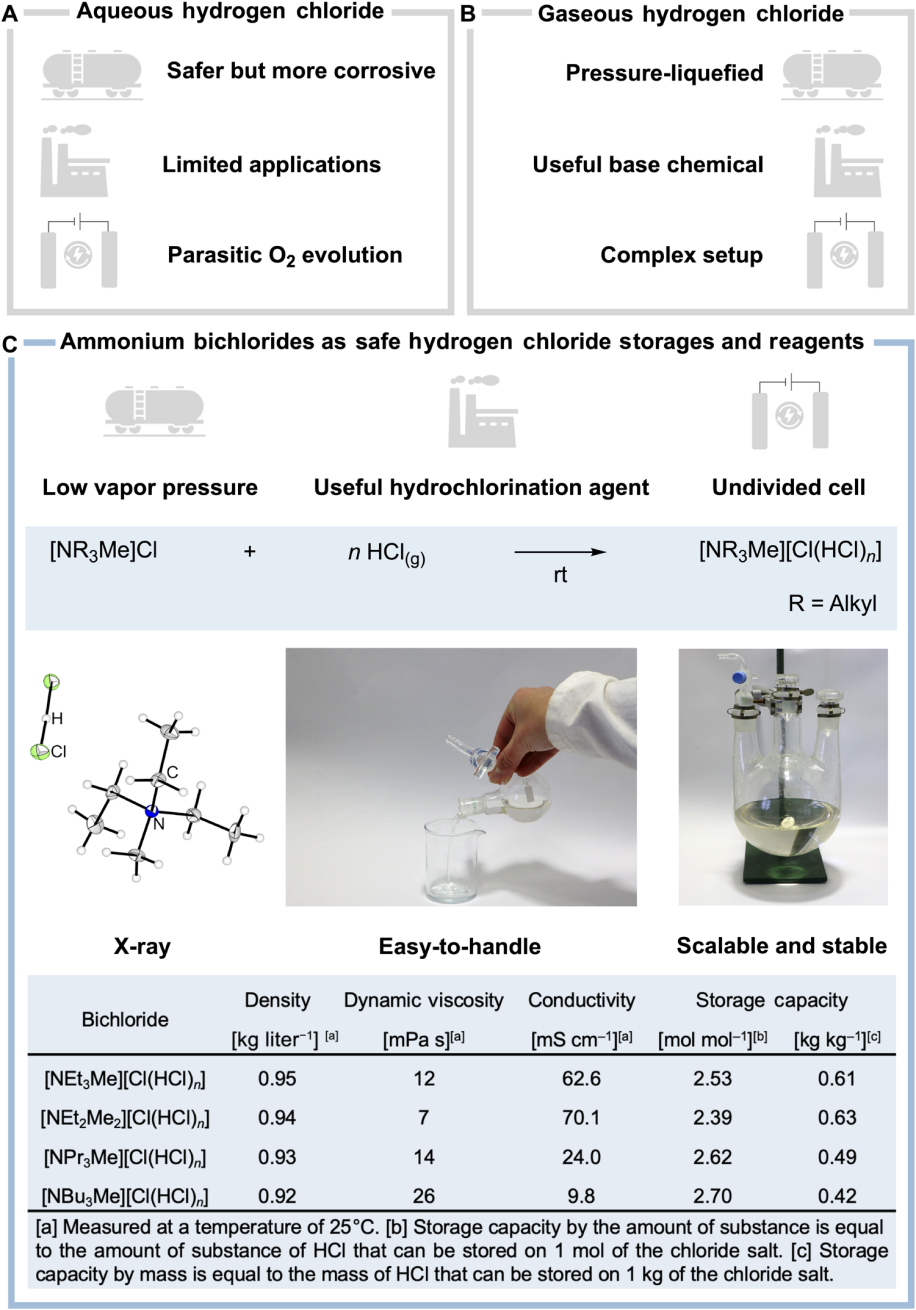
Comparison of the properties of different hydrogen chloride systems. (**A**) Aqueous hydrogen chloride. (**B**) Gaseous/liquid hydrogen chloride. (**C**) Ammonium bichlorides. rt, room temperature.

The thus-obtained HCl is either dissolved in water to form hydrochloric acid (typically with a concentration of 20%) or used directly as gaseous HCl. Although hydrochloric acid shows a higher corrosivity, it is safer to store and transport. However, it suffers from the substantial disadvantage that it can only be used for few industrial processes as most of them rely on anhydrous HCl (Fig. 1A) (*5*, *6*).

Gaseous hydrogen chloride is now stored using pressure liquefication building up a large vapor pressure of 42.6 bar absolute at 20°C (*8*). Most of the accidents related to HCl happen due to vessel or valve failures resulting in the uncontrolled release of toxic HCl and a possible rupture of the whole system (*9*). Because of these challenges, the storage of pressure-liquefied HCl in industrial plants is usually avoided particularly on large scale (Fig. 1B) (*5*, *6*). Instead, it is produced on-site and directly used in further industrial processes such as the hydrochlorination of acetylene to afford monomeric vinyl chloride (VCM) which is further manufactured to polyvinyl chloride (PVC) (*6*).

However, the strong interlocking of hydrogen chloride production and consumption emphasizes the inherent lack of flexibility of this highly interdependent process architecture. Because of the demand to decrease the dependency of the chemical industry on fossil energy sources, strategies to incorporate renewable energies are urgently needed to enable an electrification of the chemical industry (*10*, *11*). As solar or wind energies are renewable yet inherently fluctuating and thus unreliable depending on weather circumstances (dark doldrums), the flexibilization of chemical processes is of crucial importance (*12*–*14*). Therefore, innovative concepts for the storage of power and base chemicals (power-to-X) could become a key technology for a transformation of the chemical industry (*15*–*17*). In this context, a safe and industrially scalable hydrogen chloride storage system is required.

As the formation of HCl as a by-product already exceeds its industrial demand and is expected to grow in the next years, it is necessary to regenerate chlorine from HCl, for instance, by the Deacon process (*1*, *18*–*20*) or by electrolysis (*5*–*7*, *21*). Although the electrolysis of gaseous HCl is thermodynamically almost as efficient as the Deacon process, it requires special cell designs and membranes (*22*). On the other hand, the electrolysis of hydrochloric acid is an established process but suffers from the parasitic oxygen evolution by water electrolysis and an inherently higher-energy consumption due to the hydration of the Cl^−^ and H^+^ ions (*7*). Given these facts, both aqueous and gaseous HCl show substantial disadvantages that now limit the utilization of these resources.

An attractive complementary approach would be a technique that combines the superior safety profile of hydrochloric acid with the advantageous reactivity and electrochemical properties of gaseous hydrogen chloride. Although ionic liquids (*23*–*25*) and metal-organic frameworks (*26*) for the reversible absorption of HCl are reported, none of them were used as scalable storages that can directly be used for hydrochlorination reactions and electrolysis. Recently, Geng and coworkers (*27*) demonstrated that imidazole-based deep eutectic solvents can be used for the efficient coupling of HCl capture and conversion.

## RESULTS AND DISCUSSION

### Investigation of the physicochemical properties of ammonium bichloride ionic liquids

During our ongoing research on halogen chemistry in cooperation with partners from the industry, we found that triethylmethylammonium chloride, [NEt_3_Me]Cl, can be used as safe and reversible chlorine storage forming the corresponding trichloride [NEt_3_Me][Cl(Cl_2_)*_n_*] (*2*, *28*). This ionic liquid can directly be used as a chlorination reagent for the synthesis of base chemicals such as phosgene (*29*). On the basis of these works and our previous investigations of poly(hydrogen halide) halogenates (*30*), we anticipated that ammonium chloride salts [NR_3_Me]Cl could be a scalable platform for the safe and reversible storage of hydrogen chloride while serving as an easy-to-handle hydrochlorination reagent and as a feedstock for the electrochemical generation of hydrogen and chlorine.

To examine this possibility, we treated the readily available ammonium chlorides [NEt_3_Me]Cl, [NEt_2_Me_2_]Cl, [NPr_3_Me]Cl, and [NBu_3_Me]Cl with gaseous hydrogen chloride (Fig. 1C) (*30*, *31*). To elucidate the molecular structure of the formed ionic liquids, we prepared colorless crystals suitable for single-crystal x-ray diffraction by adding CH_2_Cl_2_ to the ionic liquid, before slowly cooling to −80°C (see figs. S14 to S17 and tables S5 and S6). The molecular structures in the solid state of the species [NR_3_Me][Cl(HCl)] proved the presence of a so-called bichloride anion, also known as hydrogen chloride chlorate [Cl-H-Cl]^−^ (shown for [NEt_3_Me][Cl(HCl)] in Fig. 1C).

Although an accurate determination of the position of protons by x-ray diffraction is difficult, the molecular structure in the solid states displays an almost linear geometry for the [Cl-H-Cl]^−^ anion. Our group showed previously by quantum-chemical calculations that the combination of halide anions with hydrogen halides can result in compounds with strong hydrogen bond interactions having a large positive charge at the central hydrogen atom. The main contribution of this binding energy can be described as a charge transfer. Thus, the donation of electron density from the lone pair of the chloride ion into the σ* orbital of the H-Cl bond results in a 3c-4e bond (see fig. S18) (*30*, *32*).

To further investigate this system, we analyzed the storage capacities of the ammonium chloride salts by loading them until the system reached atmospheric pressure. Notably, the four different ammonium chloride salts used can store hydrogen chloride in various quantities forming mixed systems that can be expressed by the formula [NR_3_Me][Cl(HCl)*_n_*]. More precisely, a bichloride [Cl(HCl)]^−^ is only formed in the case of a hydrogen chloride loading of *n* = 1; however, for clarity, we call all species [Cl(HCl)*_n_*]^−^ “bichlorides.” Furthermore, the densities, viscosities, and conductivities of the formed bichlorides were analyzed (Fig. 1C, bottom).

In analogy to the previously reported polychlorides (*28*), the bichlorides having ammonium cations with longer alkyl chains (e.g., butyl) show a higher dynamic viscosity but lower densities and conductivities. Notably, the bichlorides [NEt_3_Me][Cl(HCl)*_n_*] and [NEt_2_Me_2_][Cl(HCl)*_n_*] have lower viscosities; thus, they should be more suitable for pumping within industrial plants. [NEt_3_Me]Cl shows a higher storage capacity than [NEt_2_Me_2_]Cl and can be prepared from the abundant materials NEt_3_ and MeCl. Therefore, we consider [NEt_3_Me]Cl as the most promising platform for hydrogen chloride storage and focused our further investigations on this system.

As the bichloride [NEt_3_Me][Cl(HCl)*_n_*] can be readily prepared by exposing [NEt_3_Me]Cl to an atmosphere of HCl, we were able to synthesize 1 kg of [NEt_3_Me][Cl(HCl)*_n_*] demonstrating the scalability of the HCl storage (Fig. 1C and see fig. S1). Notably, we observed no decomposition of the loaded storage over a period of at least 6 months.

### Hydrogen chloride release and vapor pressure curves

**Fig. 2.**
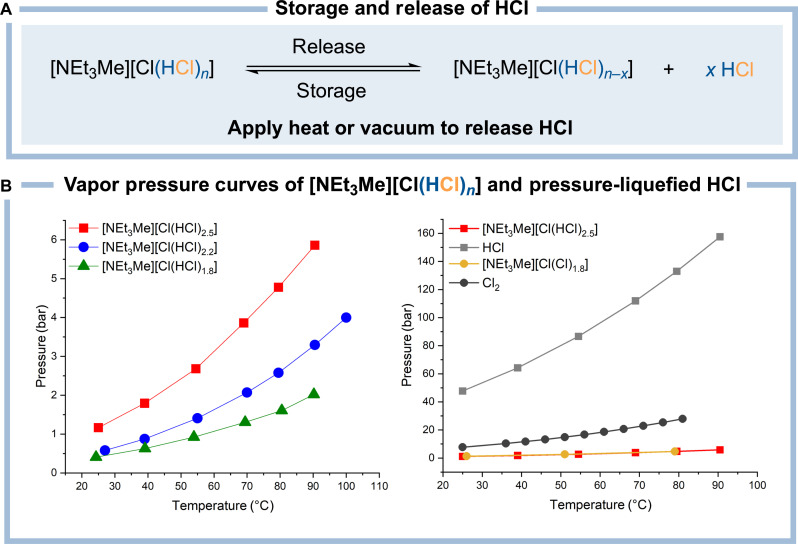
Vapor pressure of the bichloride ionic liquid in comparison to the trichloride ionic liquid, pressure-liquefied HCl, and pressure-liquefied Cl_2_. (**A**) Reversible storage and release of hydrogen chloride using the bichloride system. (**B**) The temperature-dependent HCl vapor pressure of the bichloride system dependent on the HCl loading (*n* = 1.8 to 2.5) (left) and, for comparison, the temperature-dependent vapor pressures of the trichloride, gaseous HCl, and gaseous Cl_2_ (right).

To use the here presented ionic liquid [NEt_3_Me][Cl(HCl)*_n_*] as a storage medium for HCl, its controlled release by applying heat would be desirable for industrial use (Fig. 2A). Because of the relatively low bonding energy between HCl and the chloride anion in [NEt_3_Me][Cl(HCl)*_n_*], it forms an equilibrium to the ammonium chloride [NEt_3_Me]Cl and hydrogen chloride gas. Therefore, the system shows an HCl vapor pressure depending both on the temperature and the amount of the bonded hydrogen chloride *n*. To investigate this dependency for the system [NEt_3_Me][Cl(HCl)*_n_*], its vapor pressure curves were determined (Fig. 2B and see figs. S2 and S3). As expected, the vapor pressure increases with higher values of *n* corresponding to a higher HCl loading and with higher temperatures. These measurements imply that HCl can be released from [NEt_3_Me][Cl(HCl)*_n_*] using heat or vacuum.

When comparing the bichloride with the highest storage capacity (*n* = 2.5) with pure liquid HCl, the bichloride reveals a vapor pressure of 1.17 bar, while liquid hydrogen chloride has a pressure of 47.7 bar at a temperature of 25°C (Fig. 2B, right) (*33*). This is even more pronounced at 90°C, when liquid HCl has a vapor pressure of 157 bar, but the value for [NEt_3_Me][Cl(HCl)_2.5_] rises only to 5.86 bar. Since most gas vessel failures occur due to a sharp pressure increase induced, for instance, by an unforseen temperature rise (*9*), the notable difference between the vapor pressures of [NEt_3_Me][Cl(HCl)_2.5_] and liquid HCl renders the superior safety profile of the bichloride-based hydrogen chloride storage.

### Reaction behavior of the ionic liquid [NEt_3_Me][Cl(HCl)*_n_*]

**Fig. 3.**
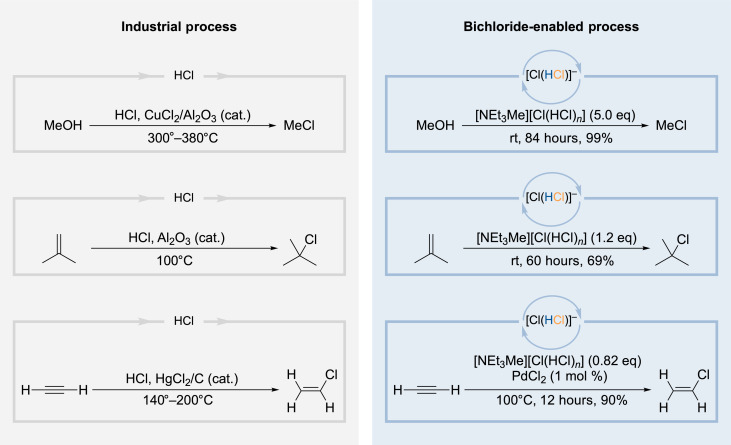
Reactions with hydrogen chloride under typical industrial conditions and by using the bichloride ionic liquid. Equivalents of the bichloride are given for the total amount of hydrogen chloride in the system.

As the release of hydrogen chloride from the storage medium requires energy, we envisioned to spare this step by using the bichloride directly as a hydrochlorination reagent for the manufacturing of industrially relevant chemicals. At the outset, we attempted the conversion of methanol (MeOH) to chloromethane (MeCl) (Fig. 3, top). With a production volume of 3.35 million tons per year, chloromethane ranks among the most important C1 building blocks in the chemical industry and is widely used, e.g., for the manufacturing of silicones and methylcellulose (*5*). Chloromethane is produced in substantial amounts by the reaction of MeOH with hydrogen chloride, which is performed at 300° to 380°C in the gas phase using CuCl_2_ on Al_2_O_3_ as catalyst (*5*). In contrast, when we treated MeOH with [NEt_3_Me][Cl(HCl)*_n_*] at room temperature, we observed the formation of chloromethane in 99% yield.

Next, we investigated the transformation of isobutylene to *tert*-butyl chloride, a chemical that is commonly used for the introduction of *tert*-butyl groups into organic molecules (Fig. 3, middle). While the industrial process is performed at 100°C using an aluminum oxide catalyst, we could realize this reaction with 1.2 equivalents of [NEt_3_Me][Cl(HCl)*_n_*] without a catalyst at room temperature in 69% yield. Notably, we observed no polymerization of isobutylene under these conditions (*34*).

For the production of PVC, the corresponding monomer vinyl chloride (VCM) is synthesized on a scale of 40 million t a^−1^ (*35*). Although vinyl chloride is primarily produced from ethylene, more recently, the increased availability of acetylene led to a resurrection of the formerly dominant production route by hydrochlorination of acetylene (13 million t a^−1^). However, in this process, mercury(ii) chloride supported on carbon is typically used. Because of the environmental problems caused by mercury, the Minamata Accord mandated a phase-out of its use in industrial processes (*35*, *36*). Intensive research revealed that gold and other transition metal catalysts can act as appropriate alternatives (*35*–*39*). Inspired by Li and coworkers (*40*), we attempted the hydrochlorination of acetylene with the bichloride [NEt_3_Me][Cl(HCl)*_n_*] in the presence of catalytic amounts of palladium(ii) chloride. After 12 hours at 100°C, vinyl chloride was obtained selectively in a yield of 90% (Fig. 3, bottom). These three industrially relevant applications demonstrate the ability of the bichloride ionic liquid to serve not only as a storage system but also its function as a readily available gaseous HCl source for selective hydrochlorination reactions under mild conditions.

### Electrochemical investigations of the bichloride ionic liquid

As now only 15% of the HCl formed as a by-product in chlorination processes are regenerated to chlorine, there is an urgent demand to develop efficient systems to recover and recycle this resource for the chlorine industry (*7*). Therefore, we envisioned to electrolyze [NEt_3_Me][Cl(HCl)*_n_*] as we anticipated to convert the HCl bound in this ionic liquid to Cl_2_ and H_2_ while the cation was expected to be electrochemically stable.

**Fig. 4.**
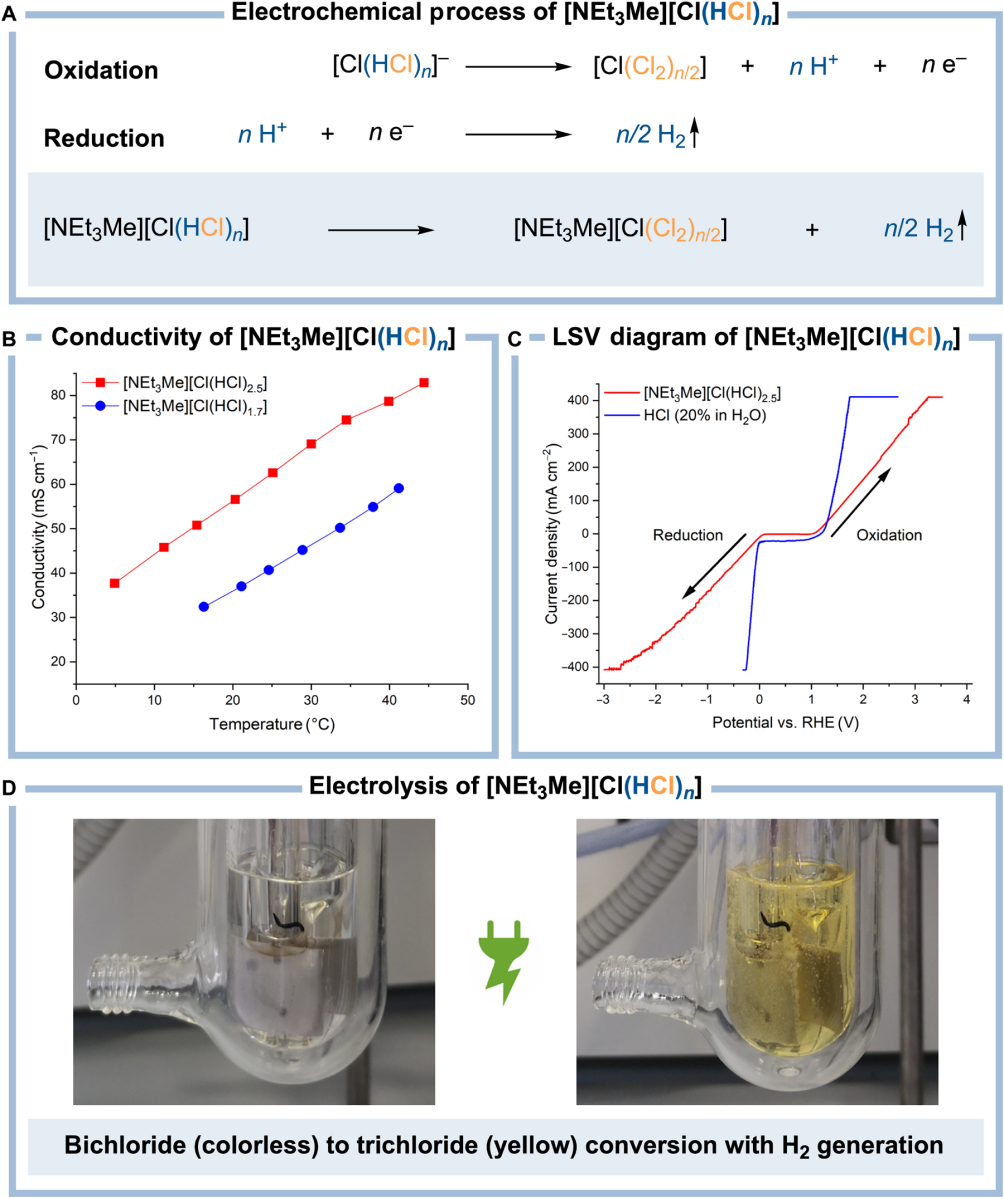
Electrolysis of the bichloride [NEt_3_Me][Cl(HCl)*_n_*]. (**A**) Electrochemical process with oxidative chlorine and reductive hydrogen production. (**B**) Temperature-dependent conductivities of the bichloride system in dependence on the HCl loading (*n* = 1.7 and 2.5). (**C**) Linear sweep voltammetry (LSV) diagram of the bichloride (blue) and 20% hydrochloric acid (red) (Pt/Pt/RHE, 100 mV s^−1^). (**D**) Electrolysis of the colorless bichloride to the yellow trichloride with H_2_ gas evolution.

Initially, we measured the conductivity of [NEt_3_Me][Cl(HCl)*_n_*] with two loadings (*n* = 1.7 and 2.5) at different temperatures, indicating that the conductivity increases both with the temperature and the HCl loading (corresponding to *n*) of the bichloride (Fig. 4B). After a brief screening of electrode materials using a two-electrode setup, we found that platinum electrodes are the most suitable, as they are showing high chemical inertness against HCl and Cl_2_ while showing the highest current densities at the lowest potentials (see table S1).

Initial linear sweep voltammetry measurements (LSV) revealed a reduction and an oxidation process of [NEt_3_Me][Cl(HCl)_2.5_] (Fig. 4C and see fig. S5 and table S2). As the applicability of reference systems in ionic liquids is still under investigation, an exact determination of the absolute potentials is only possible to a limited extent (*41*). However, it still allows determining the decomposition voltage which can be obtained from the difference between oxidation and reduction potential. For the electrolysis of [NEt_3_Me][Cl(HCl)_2.5_], a decomposition voltage of 0.98 V was obtained. In contrast, for 20% hydrochloric acid, a value of 1.23 V could be measured, which might be assigned to the electrolysis of water (*7*). Furthermore, the measurements revealed a current density of 71.16 mA cm^−2^ at 1.5 V for the oxidation of the bichloride-based system.

During the electrolysis of the bichloride [NEt_3_Me][Cl(HCl)_2.5_], we observed the formation of a colorless gas only at the cathode. According to mass spectrometry, the formed gas contains primarily hydrogen which suggests that the bichloride is electrolyzed to Cl_2_ and H_2_, but the formed Cl_2_ does not evolve as a gas to a large extent (see fig. S13). In addition, the electrolysis mixture turned rapidly from colorless to pale yellow, indicating the conversion of the colorless bichloride [NEt_3_Me][Cl(HCl)_2.5_] to the yellow trichloride [NEt_3_Me][Cl(Cl_2_)*_n_*] (Fig. 4D) (*28*). Thus, it can be assumed that the formed Cl_2_ is directly bound by the ionic liquid allowing the electrochemical conversion of [NEt_3_Me][Cl(HCl)*_n_*] to the ionic liquid [NEt_3_Me][Cl(Cl_2_)*_n_*] (Fig. 4A).

When a galvanostatic electrolysis (Pt/Pt) of [NEt_3_Me][Cl(HCl)*_n_*] was performed over 6 hours at 20°C with a constant current of 350 mA cm^−2^, a current efficiency of 30% was obtained (see fig. S6, reaction S1, and tables S3 and S4). Since no decomposition of the cation could be observed according to nuclear magnetic resonance (NMR) spectroscopy, we assume that the current efficiency is diminished by the evolution of hydrogen carrying HCl and Cl_2_ from the ionic liquid. Since the conductivity is highly dependent on the amount of bonded HCl in the ionic liquid, the loss of HCl could reduce this conductivity and thus the current efficiency of the process. As engineered processes for the electrolysis of gaseous or aqueous HCl can be realized with higher current densities and efficiencies (*6*, *7*), we expect that further optimization of electrode materials and process parameters will notably enhance the efficiency of the bichloride electrolysis.

In the bichloride electrolysis, the generated H_2_ evolves as a gas, while the formed Cl_2_ remains mostly in the ionic liquid. This offers the enormous advantage of separating both gases chemically in a simple, undivided cell. In the established electrolysis processes for gaseous and aqueous HCl, the H_2_ and the Cl_2_ generating half cells are typically separated by Nafion membranes to avoid the formation of dangerous H_2_ and Cl_2_ mixtures (*21*). However, Nafion, having a polytetrafluoroethylene (PTFE) backbone, belongs to the family of per- and polyfluoroalkyl substances (PFAS), a compound class that has caused a contamination of the environment with global dimensions (*42*, *43*). Besides this aspect, the employment of a membrane extensively increases the ohmic resistance of an electrochemical setup resulting in an increased total energy consumption (*22*). Thus, the electrolysis of HCl in the form of the ionic liquid [NEt_3_Me][Cl(HCl)*_n_*] instead of gaseous or aqueous HCl could represent a complementary approach to avoid the utilization of Nafion membranes in future. Moreover, the bichloride electrolysis can be performed under anhydrous conditions eliminating major corrosion problems associated with aqueous HCl in industrial systems while enabling the reliable and flexible on-demand generation of dry hydrogen and chlorine (*21*).

### Ionic liquids as a hub for coupled processes

**Fig. 5.**
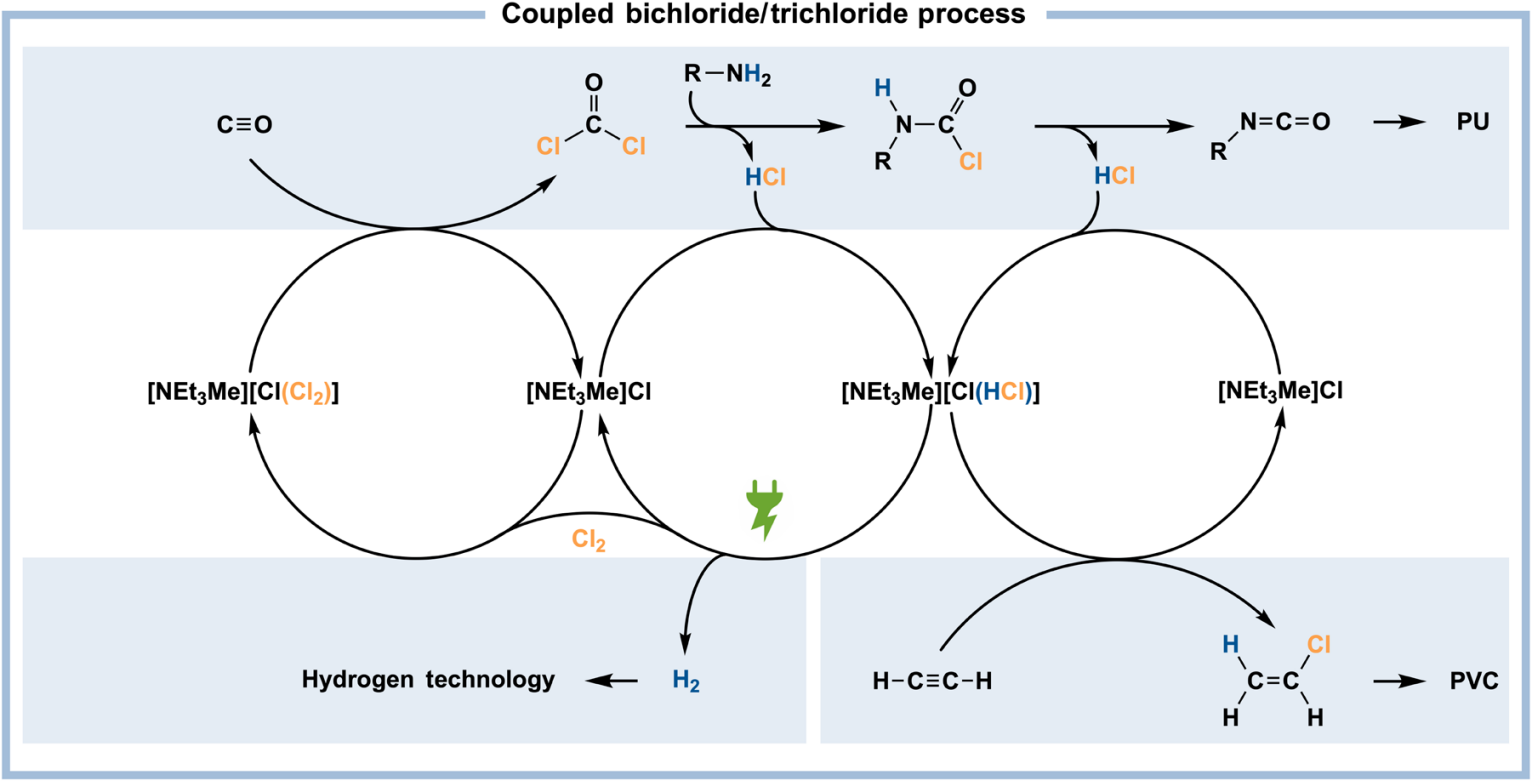
Vision of a coupled bichloride/trichloride process. The coupled process uses chlorine for the synthesis of, e.g., PU while liberating HCl that can be recycled by electrolysis or further processed using the bichloride system.

Besides these advantages, the bichloride system [NEt_3_Me][Cl(HCl)*_n_*] would release its full potential when it is coupled with the previously reported trichloride system [NEt_3_Me][Cl(Cl_2_)*_n_*] (Fig. 5). As we have recently shown, [NEt_3_Me][Cl(Cl_2_)*_n_*] can be used for the synthesis of phosgene (COCl_2_) under ambient conditions (*29*). In this reaction, the unloaded chlorine storage, [NEt_3_Me]Cl, is formed, which is essentially the precursor for both the trichloride [NEt_3_Me][Cl(Cl_2_)*_n_*] and the bichloride [NEt_3_Me][Cl(HCl)*_n_*] serving as a hub between both systems. Phosgene is primarily used for the synthesis of isocyanates that are further processed to, e.g., PU (*5*). In this process, HCl is released as a by-product that could be captured by [NEt_3_Me]Cl forming [NEt_3_Me][Cl(HCl)*_n_*], which, in turn, could either be used for the hydrochlorination of acetylene or be electrolyzed to regenerate the trichloride [NEt_3_Me][Cl(Cl_2_)*_n_*] while affording dry hydrogen. In this way, the ionic liquids [NEt_3_Me][Cl(HCl)*_n_*] and [NEt_3_Me][Cl(Cl_2_)*_n_*] sharing the same precursor, [NEt_3_Me]Cl, could be coupled to serve as a platform for the safe storage and processing of the inherently dangerous but indispensable base chemicals HCl and Cl_2_.

In conclusion, we have presented an ionic liquid-based system for the safe storage and utilization of HCl complementing the established systems of gaseous and aqueous HCl. By exposing the ammonium salts [NR_3_Me]Cl to an atmosphere of HCl, the corresponding bichlorides [NR_3_Me][Cl(HCl)*_n_*] are formed, as proven by x-ray crystallography. Among them, the ionic liquid [NEt_3_Me][Cl(HCl)*_n_*] shows a good compromise between viscosity (12 mPa s), conductivity (62.6 mS cm^−1^), and HCl storage capacity (0.61 kg kg^−1^) while using the commercially available [NEt_3_Me]Cl. By applying heat or vacuum, the stored HCl is released from [NEt_3_Me][Cl(HCl)*_n_*], regenerating the empty storage [NEt_3_Me]Cl. Instead of unloading this easy-to-handle HCl storage, it can also be used as a hydrochlorination reagent for the synthesis of the industrially important chemicals chloromethane, *tert*-butyl chloride, and vinyl chloride. Alternatively, it is possible to electrolyze the bichloride [NEt_3_Me][Cl(HCl)*_n_*] under anhydrous conditions in an undivided cell forming the corresponding chlorine-containing ionic liquid [NEt_3_Me][Cl(Cl_2_)*_n_*] and dry hydrogen. This work could open up avenues for a coupled bichloride-trichloride process, using the trichloride for chlorination reactions and the bichloride to capture and further process HCl generated as a by-product. The application of the bichloride [NEt_3_Me][Cl(HCl)*_n_*] for further industrial processes and the optimization of its electrolysis are now under investigation in our laboratories.

## MATERIALS AND METHODS

### Apparatus and materials

All substances sensitive to water and oxygen were handled under an argon atmosphere using standard Schlenk techniques and an oil pump vacuum up to 10^−3^ mbar. [NEt_2_Me_2_]Cl and [NPr_3_Me]Cl were prepared according to literature procedures (*44*). All other chemicals were obtained from commercial suppliers and were used without further purification. The salts were dried in vacuo at 100°C for 1 hour to 1 day before use, while all solvents were obtained anhydrous by storage over activated 3-Å molecular sieves. NMR spectra were recorded on a 400-MHz ECS or ECZ spectrometer by JEOL. X-ray diffraction data were collected on a Bruker D8 Venture complementary metal-oxide semiconductor area detector (Photon 100) diffractometer with Mo_Kα_ radiation. Single crystals were coated with perfluoroether oil at low temperatures around −80°C and mounted on a 0.1- to 0.2-mm Micromount. The structures were solved with the ShelXT structure solution program using intrinsic phasing and refined with the ShelXT refinement package using least squares on weighted F2 values for all reflections using OLEX2 (*45*–*47*). Hydrogen atoms were treated using the HFIX 23 (CH_2_ groups) and HFIX 137 (CH_3_ groups) restrains as implemented in ShelXL. Viscosities were measured using an Ubbelohde viscosimeter. Densities were measured using a pressure-stable Schlenk tube with a volume scale in which a defined volume of the substance was weighed. Vapor pressure determinations above 1 bar were performed in a glass autoclave (tinyclave) by BüchiGlasUster and were measured using a Solid Sense II pressure sensor from Brooks Instruments. Conductivities were measured in a temperature-controlled flask using a conductivity cell in addition to a SevenCompact Conductivity S230Meter by M. Toledo.

### General procedure for the preparation of [Cation][Cl(HCl)*_n_*]

The used bichlorides [Cation][Cl(HCl)*_n_*] (Cation = [NEt_3_Me]^+^, [NEt_2_Me_2_]^+^, [NPr_3_Me]^+^, [NBu_3_Me]^+^) were prepared by two different methods depending on their further use.

#### General procedure 1

Stoichiometric bichlorides [Cation][Cl(HCl)] were obtained by condensing the calculated stoichiometric amount of HCl onto the dried ammonium chloride salt. The addition of 0.5 ml of CH_2_Cl_2_ resulted in a clear solution from where colorless crystals (figs. S13 to S16) of [NEt_3_Me][Cl(HCl)], [NEt_2_Me_2_][Cl(HCl)], [NPr_3_Me][Cl(HCl)], and [NBu_3_Me][Cl(HCl)] were obtained by slowly cooling the solution to −80°C.

#### General procedure 2

Bichlorides with an excess of HCl were produced by placing the ammonium chloride salt in a flask, drying, and adding gaseous HCl until the system showed a total pressure of 1 bar. This procedure was supported by stirring or shaking the reaction mixture. All bichlorides [Cation][Cl(HCl)*_n_*] (Cation = [NEt_3_Me]^+^, [NEt_2_Me_2_]^+^, [NPr_3_Me]^+^, [NBu_3_Me]^+^) were obtained as colorless liquids.

#### Synthesis of [NEt_3_Me][Cl(HCl)] to determine the reaction completion time

The stoichiometric bichloride [NEt_3_Me][Cl(HCl)] was synthesized according to general procedure 2, using [NEt_3_Me]Cl (7.89 g, 52.0 mmol, 1.00 equiv.) and a determined amount of HCl (1.90 g, 52.0 mmol, 1.00 equiv.). The reaction time for the formation of the bichloride was measured to be 5 min at room temperature.

#### Kilogram synthesis of [NEt_3_Me][Cl(HCl)_n_]

The bichloride [NEt_3_Me][Cl(HCl)*_n_*] (1.00 kg, 4.12 mol) was synthesized according to general procedure 2. The ammonium chloride salt (0.625 kg, 4.12 mol, 1.0 equiv) was placed in a 2-liter three-neck flask and dried in vacuo overnight at 100°C. The flask was allowed to cool to room temperature and was connected to a hydrogen chloride gas bottle using standard Schlenk techniques. The salt was loaded with gaseous HCl by using an additional gas valve attached to the gas bottle to ensure a controlled gas release. Since the bichloride shows very high viscosities until loaded with 1.5 equivalents of HCl, it was carefully shaken from time to time until stirring was possible (fig. S1). The colorless ionic liquid was loaded with gaseous HCl (0.375 kg, 10.3 mol, 2.5 equiv) until it showed a vapor pressure of 1 bar.

Disposal of the bichloride: If necessary, the bichloride can be diluted with water to obtain hydrochloric acid and can be discarded after careful neutralization with sodium bicarbonate.

### Determination of storage capacities

A defined amount of the ammonium chloride salt of interest was placed into a pressure-stable reaction tube. The correspondent bichloride was prepared according to general procedure 2. The amount of stored hydrogen chloride was investigated by determining the mass difference between the unloaded chloride salt and the bichloride.

### Hydrogen chloride release experiments

[NEt_3_Me][Cl(HCl)_2.5_] (24.03 g, 98.89 mmol, 247.7 mmol HCl bound) was prepared in a Schlenk flask according to general procedure 2. The flask was equipped with a bubbler and was heated to 80°C for 1 hour. The released amount of HCl was measured by determining the mass difference before and after heating. A total of 3.038 g of HCl (83.32 mmol, 34%) was released after 1 hour.

In a second experiment, [NEt_3_Me][Cl(HCl)_2.7_] (24.59 g, 98.89 mmol, 262.3 mmol HCl bound) was prepared according to general procedure 2. The sample was heated to 60°C in vacuo for 7.5 hours. A total of 8.58 g of HCl (235.2 mmol, 90%) was released after 7.5 hours.

### Vapor pressure determination of [NEt_3_Me][Cl(HCl)*_n_*]

The vapor pressure of [NEt_3_Me][Cl(HCl)*_n_*] for different values of *n* was analyzed by two different methods.

#### Method 1

[NEt_3_Me]Cl (10 g, 66 mmol) was added into a 50-ml flask, dried at 100°C, and loaded with hydrogen chloride until a pressure of 1000 mbar was achieved in the system. The amount of stored hydrogen chloride was determined by weighing the flask. By applying vacuum to the stirred sample for a short time, some hydrogen chloride was removed, changing its stored amount and thus the value of *n* of the bichloride [NEt_3_Me][Cl(HCl)*_n_*]. After the system was equilibrated, the flask was weighed to determine the removed amount of HCl. The flask was cooled to a constant temperature of 15°C using a water bath, and the pressure of the system was measured (fig. S2).

#### Method 2

Dried [NEt_3_Me]Cl (10 g, 66 mmol) was added to a glass autoclave and loaded with hydrogen chloride until a system pressure of 1000 mbar was achieved. The vapor pressure of the system was determined for different temperatures, which were controlled by placing the autoclave into an oil bath (fig. S3). Measurements for different values of *n* were realized by using the method 1.

### Synthesis of chloromethane using [NEt_3_Me][Cl(HCl)*_n_*]

[NEt_3_Me][Cl(HCl)*_n_*] (0.53 g, 2.2 mmol, 2 equiv) was synthesized accordingly to general procedure 1 using [NEt_3_Me]Cl (0.33 g, 2.2 mmol, 2 equiv) and gaseous HCl (0.20 g, 5.5 mmol, 5.0 equiv). Subsequently, anhydrous and degassed MeOH (35 mg, 1.1 mmol, 1 equiv) was condensed onto the frozen, prior prepared bichloride. After slowly warming to room temperature, the reaction mixture was stirred for 84 hours. The reaction products were obtained by distilling the volatile constituents in vacuo using cooling traps held at −70°C (MeOH), −140°C (MeCl), and −198°C (HCl). All components were identified by their gas-phase infrared (IR) spectra (fig. S7), whereas the MeCl fraction was also transferred into a pressure-stable Schlenk tube to obtain its mass by weighing (56 mg, 1.1 mmol, 99%). The spectroscopic data are consistent with those in the literature (*48*).

MeCl: IR (FTIR), $\tilde{\nu }$ = 3071, 3046, 2980, 2952, 2889, 2865, 1495, 1423, 1367, 1339, 1019, 746, 732, and 714 cm^−1^.

### Synthesis of *tert*-butyl chloride using [NEt_3_Me][Cl(HCl)*_n_*]

[NEt_3_Me][Cl(HCl)*_n_*] (24 g, 0.10 mol, 0.5 equiv) was synthesized according to general procedure 1 using [NEt_3_Me]Cl (15 g, 0.10 mol, 0.5 equiv) and gaseous HCl (9.2 g, 0.25 mol, 1.2 equiv). Subsequently, gaseous isobutylene (12 g, 0.21 mol, 1.0 equiv) was condensed onto the frozen bichloride. After slowly warming to room temperature, the reaction mixture was stirred for 60 hours, resulting in the precipitation of colorless solid. The crude product was distilled at 46°C and 1013 mbar to obtain *tert*-butyl chloride as a colorless liquid (14 g, 0.15 mol, 69%). The obtained product was characterized by gas-phase IR and NMR spectroscopy (figs. S8, S10, and S11). The spectroscopic data are consistent with those in the literature (*48*, *49*).

tBuCl: IR (FTIR), $\tilde{\nu }$ = 2992, 2981, 2957, 2937, 1480, 1461, 1384, 1374, 1365, 1246, 1161, 1155, 818, 810, 592, 584, and 577 cm^−1^.

tBuCl: ^1^H NMR (400 MHz, CDCl_3_, 21°C), δ = 1.67 [s, 9H, C(CH_3_)_3_] ppm.

tBuCl: ^13^C NMR (101 MHz, CDCl_3_, 21°C), δ = 67.6 [s, 1C, C(CH_3_)_3_] and 34.6 (s, 3C, CH_3_) ppm.

### Synthesis of vinyl chloride using [NEt_3_Me][Cl(HCl)*_n_*]

[NEt_3_Me][Cl(HCl)_2.5_] (0.90 g, 3.7 mmol, 0.33 equiv) was synthesized according to general procedure 2 using [NEt_3_Me]Cl (0.56 g, 3.7 mmol, 0.33 equiv) and gaseous HCl (0.34 g, 9.0 mmol, 0.82 equiv). Subsequently, PdCl_2_ (16 mg, 0.093 mmol, 1 mol %) was added to the previously prepared bichloride before gaseous acetylene (0.29 g, 11 mmol, 1.0 equiv.) was condensed onto the frozen reaction mixture. After slowly warming to room temperature, the reaction mixture was heated to 100°C and stirred for 12 hours, resulting in the formation of a colorless solid. The reaction products were obtained by distilling the volatile constituents in vacuo using cooling traps held at −140°C [Vinyl chloride (VCM)] and −198°C (HCl, C_2_H_2_). All components were identified by their gas-phase IR spectra (fig. S9), whereas the pressure of the vinyl chloride fraction was also determined in a defined volume to obtain its mass by the ideal gas law (0.63 g, 0.010 mol, 90%). The spectroscopic data are consistent with those in the literature (*48*).

VCM: IR (FTIR), $\tilde{\nu }$ = 3129, 3095, 3075, 1884, 1844, 1804, 1786, 1622, 1598, 1521, 1504, 1381, 1358, 1289, 1269, 1041, 1022, 963, 942, 923, 898, 730, 715, 636, 618, and 603 cm^−1^.

### Electrochemical investigations

#### Electrode material experiments

To investigate the applicability of the electrochemical oxidation of [NEt_3_Me][Cl(HCl)_2.5_], initial LSV scans for electrode screening were conducted in oven-dried glassware with a two-electrode setup with a BioLogic SP-300 potentiostat (100 pA to 2 A, ±10 V), using platinum foil electrodes (Chempur, 0.5 mm by 5 mm by 120 mm, 99.9%), tantalum wire electrodes (diameter of 1 mm, 120 mm, 99.9%), polished graphite electrodes (Covestro, 5 mm by 9 mm by 35 mm), and boron-doped diamond electrodes (Condias, DIACHEM electrode type, 70 by 10 by 3). Platinum foil, polished graphite, and boron-doped diamond electrodes were used with an immersed surface area of 0.75 cm^2^ (5 mm by 15 mm), and tantalum wire electrodes were immersed at a depth of 15 mm (table S1). The different electrode materials were compared by their onset potentials and their current densities at an overpotential of 0.5 V higher than the onset potential. The lowest onset potential was attained by using platinum electrodes showing also high chemical resistance against the used ionic liquids. Thus, all further electrochemical experiments were conducted using platinum electrodes.

#### Electrochemical cell and electrode setup

Before any electrochemical reaction, the glass reactor was dried at 120°C for 12 hours. All platinum electrodes (99.99%, 10 mm by 10 mm by 0.5 mm) were welded (tungsten inert gas welding) to the same metal wire with a diameter of 1 mm on their backside. Before any reaction, all polished electrodes were cleaned with acetone, dried with compressed air, and then fixed in the electrode cover using a PTFE sealing tape. All electrode wires were covered with PTFE tubing to prevent contact with the reaction compounds. An H-spacer made of glass was placed between working and counter electrode to enable a planar parallel position and to ensure a consistent distance of 15 mm between both electrodes.

#### Experimental setup for electrochemical investigations

The following setup was used for all electrochemical experiments, which were performed in an undivided cell under inert conditions (fig. S4) at a temperature of 25°C and a constant electrode distance of 15 mm. The electrodes were mounted to the electrochemical reactor (cf. the "Electrochemical cell and electrode setup" section) which was further equipped with a stirring bar and water cooling. Afterward, the Luggin capillary was filled with the compound of interest [12 ml of [NEt_3_Me][Cl(HCl)_2.5_] or hydrochloric acid (20% HCl in H_2_O)] and also mounted to the cell. A reversible hydrogen electrode (RHE) (Hydroflex from Gaskatel GmbH) was used as a reference electrode and fixed inside the Luggin capillary. The electrochemical reactor was then filled with the reactant [20 ml of [NEt_3_Me][Cl(HCl)_2.5_] or hydrochloric acid (20% HCl in H_2_O)]. The reactant was stirred, and all electrodes were connected to an Ivium CompactStat 5A potentiostat.

#### Electrochemical methods

All electrochemical measurements were recorded with an Ivium CompactStat 5A potentiostat, whereas data analysis was carried out using the IviumSoft software.

#### Linear sweep voltammetry methods

LSV methods (table S2 and fig. S5) were conducted by applying the potential against the used RHE.

#### Chronopotentiometry methods

The chronopotentiometric bulk electrolysis method was performed on the basis of the ideal time needed to convert [NEt_3_Me][Cl(HCl)_2.5_] (20 ml) to a 2.0 mmol/ml solution of the trichloride in [NEt_3_Me][Cl(HCl)_2.5–*n*_(Cl_2_)_*n*/2_] quantitatively at a constant current of −350 mA for 21,600 s (6 hours) with an electrode surface of 1 cm^2^ under the assumption of a total conversion by the transfer of two electrons and 100% selectivity. Samples of the reaction mixture for NMR measurements were taken before the electrolysis and at a constant time interval of 3600 s (1 hour) (seven samples in total).

#### Chemical analysis of the reaction mixture

To determine the current efficiency of the electrolysis, the chlorine content of the reaction mixture was determined by iodometric titration (see the Supplementary Materials) (*50*).

## Supplementary Materials

### This PDF file includes:

Supplementary Materials and Methods Figs. S1 to S18 Tables S1 to S6 Legend for movie S1 References
