# Supplementary material for: Bichloride-based ionic liquids for the merged storage, processing, and electrolysis of hydrogen chloride
Source: Sci Adv. 2024 Apr 3;10(14):eadn5353. doi: 10.1126/sciadv.adn5353 (PMC10990271; doi:10.1126/sciadv.adn5353)
Supplement: Supplementary file 1 — Supplementary Materials and Methods Figs. S1 to S18 Tables S1 to S6 Legend for movie S1 References [file sciadv.adn5353_sm.pdf]

Science Advances
AAAS

Supplementary Materials for

Bichloride-based ionic liquids for the merged storage, processing, and electrolysis of hydrogen chloride

Gesa H. Dreyhsig *et al.*

Corresponding author: Sebastian Riedel, s.riedel@fu-berlin.de

Sci. Adv. **10**, eadn5353 (2024)
DOI: 10.1126/sciadv.adn5353

The PDF file includes:

Supplementary Materials and Methods
Figs. S1 to S18
Tables S1 to S6
Legend for movie S1
References

Other Supplementary Material for this manuscript includes the following:

Movie S1

General procedure for the preparation of [Cat][Cl(HCl)$_n$]

Kilogram synthesis of [NEt$_3$Me][Cl(HCl)$_n$]

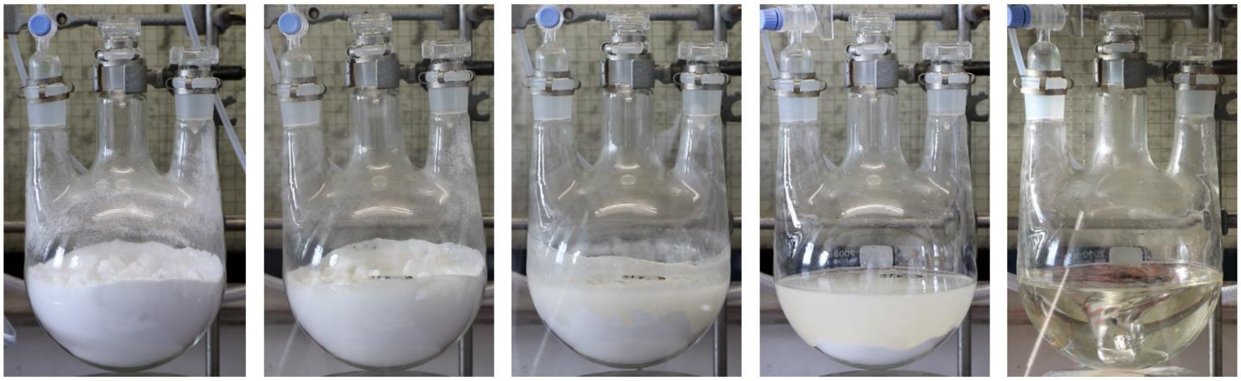

Fig. S1. Pictures of the synthesis of the bichloride [NEt$_3$Me][Cl(HCl)$_n$] by loading the ammonium salt with gaseous hydrogen chloride with increasing HCl content from left to right.

Vapor pressure determination of [NEt$_3$Me][Cl(HCl)$_n$]

The vapor pressure of [NEt$_3$Me][Cl(HCl)$_n$] for different values of *n* was analyzed by two different methods:

Method 1

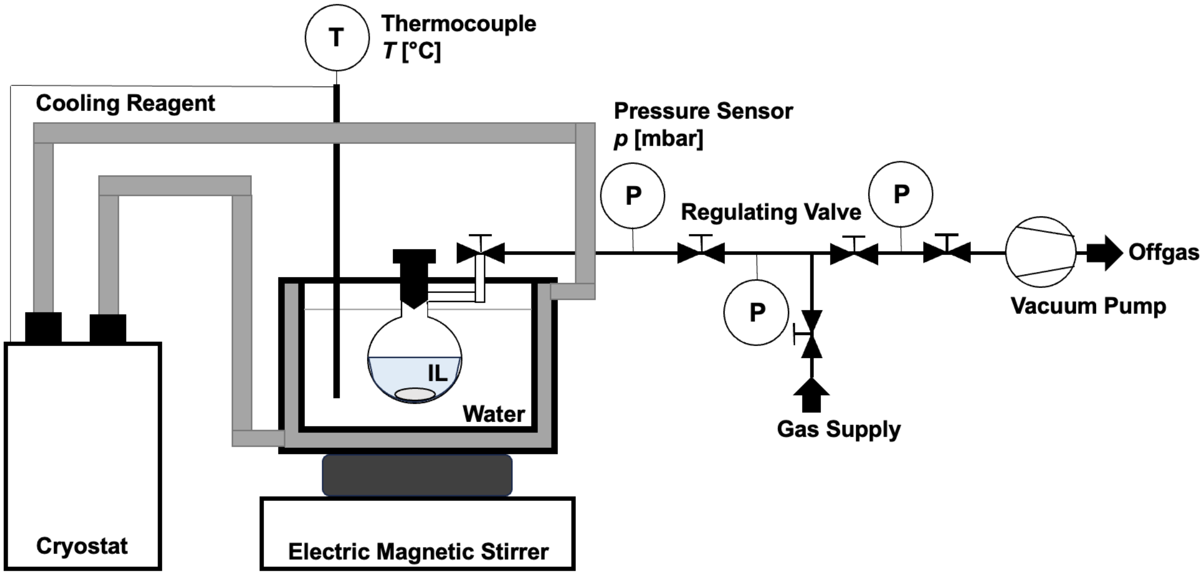

Fig. S2. Schematic representation of the used equipment for the determination of the vapor pressure curve of [NEt$_3$Me][Cl(HCl)$_n$] for different values of *n* (method 1).

Method 2

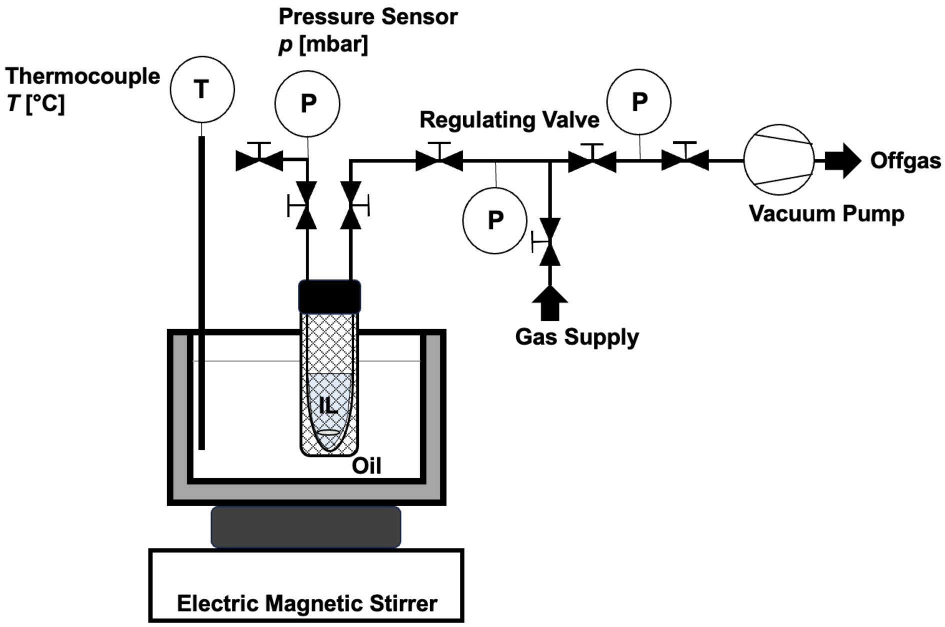

Fig. S3. Schematic representation of the used equipment for the determination of the vapor pressure curve of $[NEt_3Me][Cl(HCl)_n]$ at different temperatures for different values of *n* (method 2).

Electrochemical investigations

Electrode material experiments

Table S1. Electrode material measurements at a 6 mm distance

Material	Onset Potential [V]	Current Density at overpotential +0.5 V above Onset [mA cm^{-2}]
**Platinum**	1.004	98.5
**Tantalum**	1.679	9.5
**Boron-doped Diamond**	1.859	21.9
**Graphite**	1.938	22.0

Experimental setup for electrochemical investigations

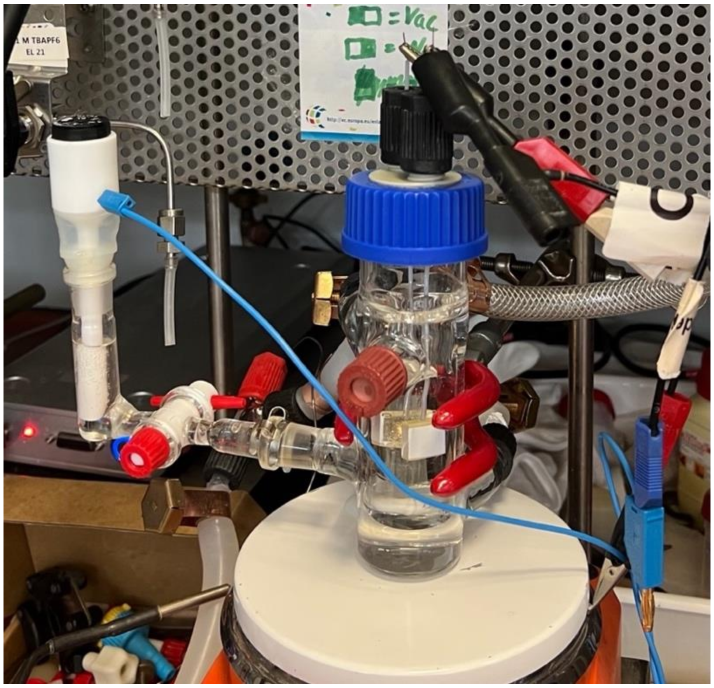

Fig. S4. Experimental setup of the undivided cell used for the electrolysis of [NEt_3Me][Cl(HCl)$_{2.5}$].

Electrochemical methods

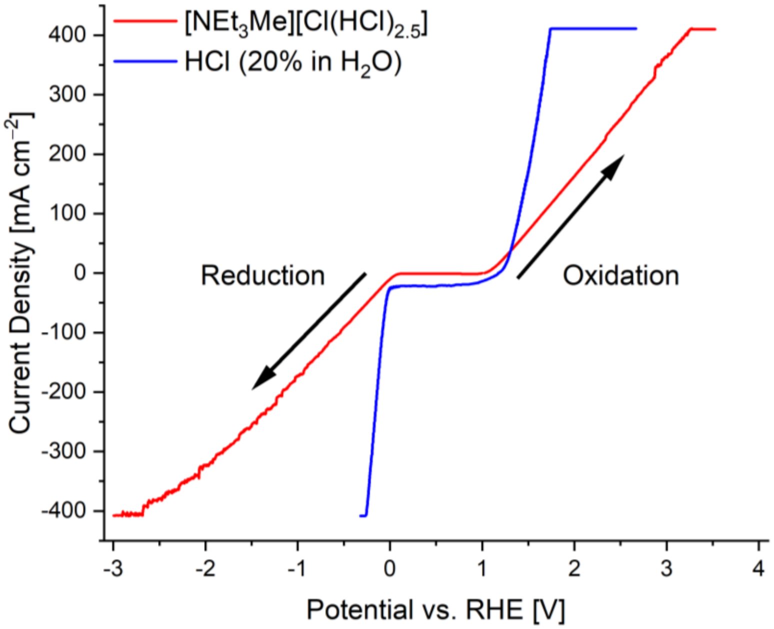

Fig. S5. Attached linear sweep voltage diagram of the bichloride [NEt_3Me][Cl(HCl)$_{2.5}$] (blue) and 20% hydrochloric acid.

Table S2. Electrochemical linear sweep voltammetry methods

**Compound**	**Start Potential [V] vs. RHE**	**End Potential [V] vs. RHE**	**Scan Rate [mV s^{-1}]**
**[NEt_3Me][Cl(HCl)$_{2.5}$]**	1	3.5	100
	1	–3.0	100
**20% HCl in H_2O**	0.7	2.2	100
	0.7	–0.8	100

<u>Chemical analysis of the reaction mixture</u>

To determine the current efficiency of the electrolysis, the chlorine content of the reaction mixture was determined by iodometric titration (**Reaction S1**) (*50*). Therefore, after electrolysis for 6 h a sample of 10 mL (total volume of the reaction mixture = 22 mL) of the reaction mixture was quenched using a 4 M aq. NaOH. To prevent chlorine loss by quenching, the reaction mixture was introduced into a glass cylinder with a length of 1 m and a diameter of 2.5 cm filled with 4 M aq. NaOH to enable long contact times (**Fig. S6**).

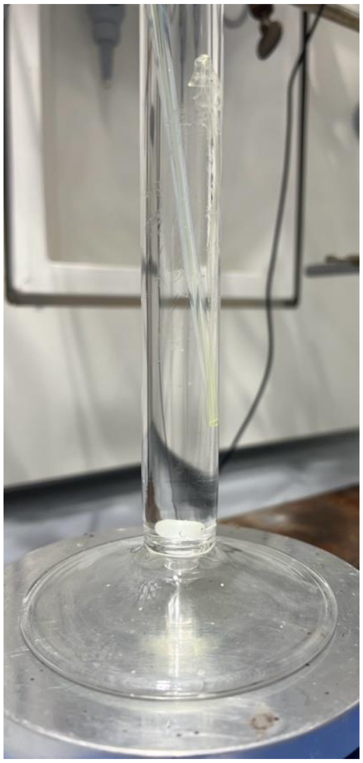

Fig. S6. Quenching of the [NEt_3Me][Cl(HCl)$_{2.5-n}$(Cl_2)$_{n/2}$] mixture after the electrolysis of [NEt_3Me][Cl(HCl)$_{2.5}$] with 4 M aq. NaOH in a glass cylinder.

An excess of KI (12 g, 72 mmol) was added, and the obtained solution was stirred until the initial yellow color vanished. The mixture was transferred into a volumetric flask and diluted with water to obtain a total volume of 500 mL. Samples of 20 or 50 mL were taken, acidified using

25% aq. HCl (pH = 1 – 2) and the concentration of iodine was determined by titration using 0.1 M aq. $Na_2S_2O_3$ (titer = 1.015). To improve the sensitivity of the method, a starch solution was added to the iodine solution. Several reactions are taking place during the determination of the concentration (**Reaction S1**).

$$[NEt_3Me][Cl(HCl)_{2.5\text{-}x}(Cl_2)_{x/2}] + 2.5\ NaOH_{(aq)} \longrightarrow [NEt_3Me]Cl + 2.5\text{-}x/2\ NaCl + x/2\ NaOCl + 2.5\ H_2O$$

$$NaOCl + 2\ KI + H_2O \longrightarrow NaCl + I_2 + 2\ KOH$$

$$3\ I_2 + 6\ NaOH \longrightarrow 5\ NaI + NaIO_3 + 3\ H_2O$$

$$5\ NaI + NaIO_3 + 6\ HCl \longrightarrow 3\ I_2 + 6\ NaCl + 3\ H_2O$$

$$I_2 + 2\ Na_2S_2O_3 \longrightarrow 2\ NaI + Na_2S_4O_6$$

Reaction S1. Reactions take place during the iodometric determination of the chlorine concentration. *x* = conversion factor.

The following amount of substance of Cl_2 was determined after the electrolysis of 22 mL $[NEt_3Me][Cl(HCl)_{2.5}]$ for 6 h with a constant current of 350 mA (**Tab. S3**).

Table S3. Determined amount of substance of Cl_2 present in the reaction mixture, $V_{titration}(I_2)$ = 50 mL

$V_{titration}(S_2O_3^{2-})$ [mL]	$n_{titration}(S_2O_3^{2-})$ [mmol]	$n_{titration}(Cl_2)$ [mmol]	$n_{sample}(Cl_2)$ [mmol]	$n_{total}(Cl_2)$ [mmol]
10.2	1.04	0.520	5.20	11.4
10.2	1.04	0.520	5.20	11.4

To estimate the error of the method, a sample of $[NEt_3Me][Cl(HCl)_{2.34}(Cl_2)_{0.1}]$ was prepared by loading $[NEt_3Me]Cl$ (10 g, 66 mmol, 1.0 equiv) into a pressure stable flask and adding HCl (5.63 g, 0.155 mol, 2.34 equiv) and Cl_2 (0.480 g, 6.77 mmol, 0.10 equiv) by condensation to achieve a total volume of 14 mL. The Cl_2 concentration of the sample was determined following the procedure described above (**Tab. S4**).

Table S4. Determined amount of substance of Cl_2 present in the reaction mixture. $V_{titration}(I_2)$ = 50 mL

$V_{titration}(S_2O_3^{2-})$ [mL]	$n_{titration}(S_2O_3^{2-})$ [mmol]	$n_{titration}(Cl_2)$ [mmol]	$n_{sample}(Cl_2)$ [mmol]	$n_{total}(Cl_2)$ [mmol]
7.6	0.771	0.386	3.86	5.40
7.7	0.776	0.388	3.88	5.43

The determined value for the amount of chlorine (5.415 mmol) corresponds to only 80% of the actual amount of chlorine present in the prepared system (6.77 mmol). This indicates that significant amounts of chlorine are lost during the quenching procedure and the error is estimated to be 20%. The theoretic amount of substance that can be generated during an electrolysis experiment performed with a constant current can be calculated according to the following formula (*n* = amount of substance, *I* = current, *t* = time, *z* = amount of electrons needed for the electrolysis of mol of molecules (for generation of Cl_2 from HCl *z* = 2), *F* = Faraday constant).

$$n_{theory}(Cl_2) = \frac{It}{zF} = \frac{0.35\ \text{A} \cdot 21600\ \text{s}}{2 \cdot 96485.3\ \text{As mol}^{-1}} = 39.1\ \text{mmol}$$

To determine the current efficiency of the electrolysis the obtained amount of substance after the electrolysis can be compared to the calculated amount of substance:

$$\beta = \frac{n_{total}(Cl_2)}{n_{theory}(Cl_2)} = \frac{11.4\ \text{mmol}}{39.1\ \text{mmol}} = (29.1 \pm 5.8)\%$$

IR Spectra

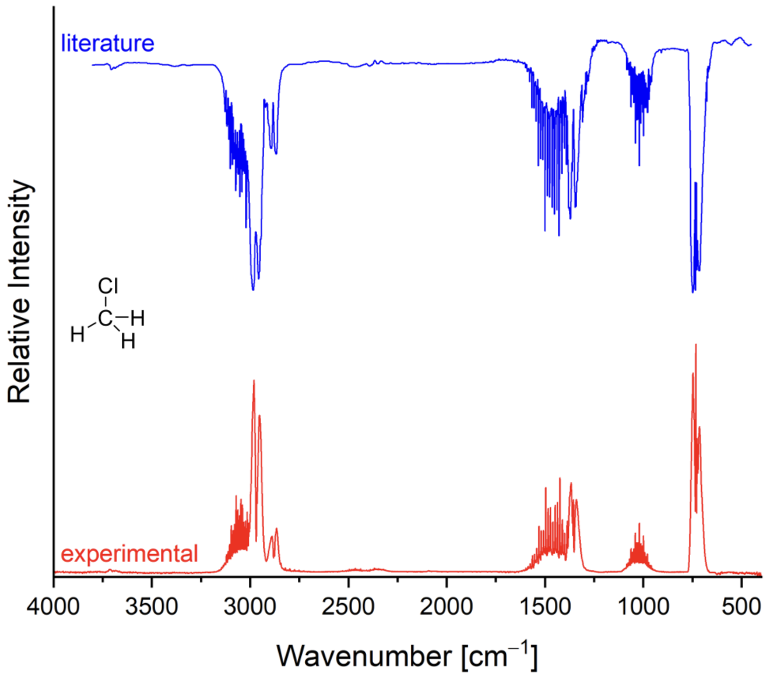

Fig. S7. IR spectrum of the purified product of the reaction of methanol with $[NEt_3Me][Cl(HCl)_{2.5}]$ (red) and the corresponding literature spectrum (*48*) of chloromethane (MeCl) (blue).

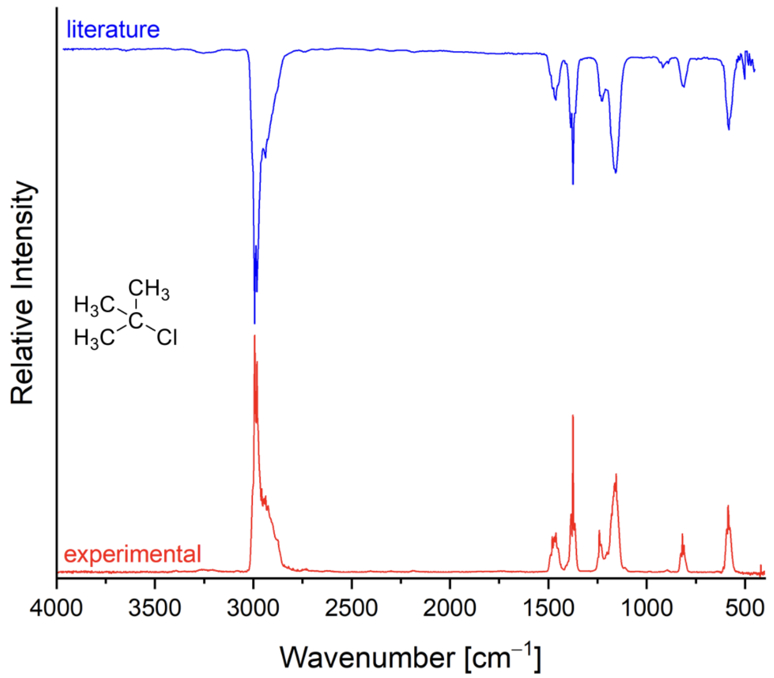

Fig. S8. IR spectrum of the purified product of the reaction of isobutylene with [NEt_3Me][$Cl(HCl)_{2.5}$] (red) and the corresponding literature spectrum (*48*) of *tert*-butyl chloride (tBuCl) (blue).

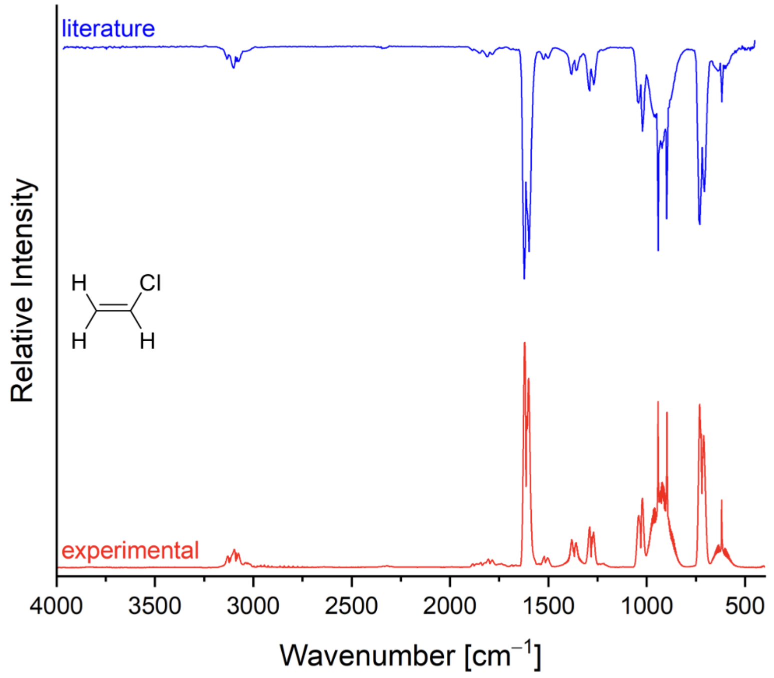

Fig. S9. IR spectrum of the purified product of the reaction of acetylene with [NEt_3Me][$Cl(HCl)_{2.5}$] (red) and the corresponding literature spectrum (*48*) of vinyl chloride (VCM) (blue).

NMR Spectra

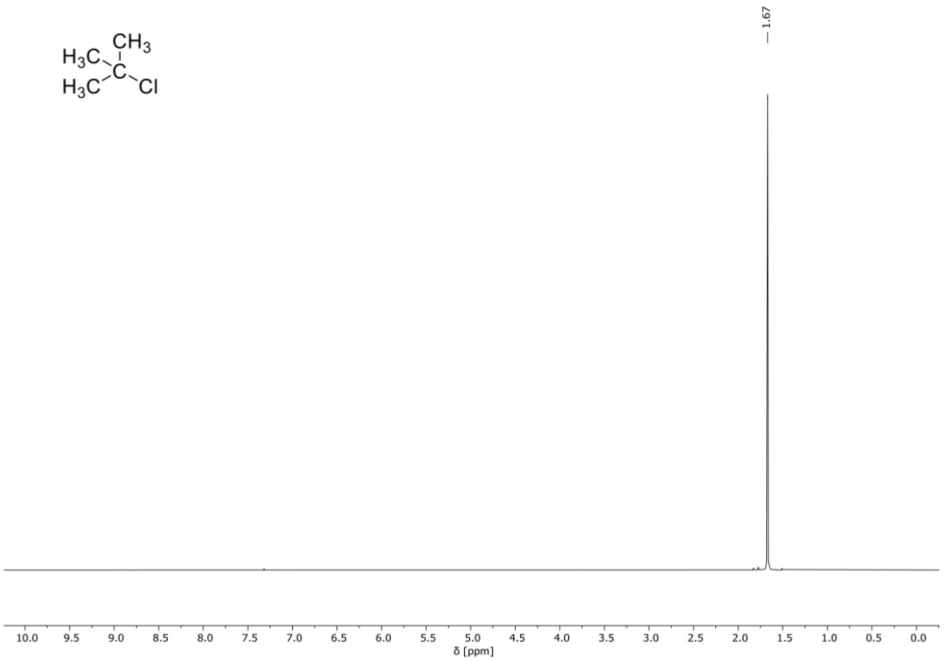

Fig. S10. ^{1}H NMR (400 MHz, $CDCl_3$, 21 °C) spectrum of *tert*-butyl chloride (tBuCl).

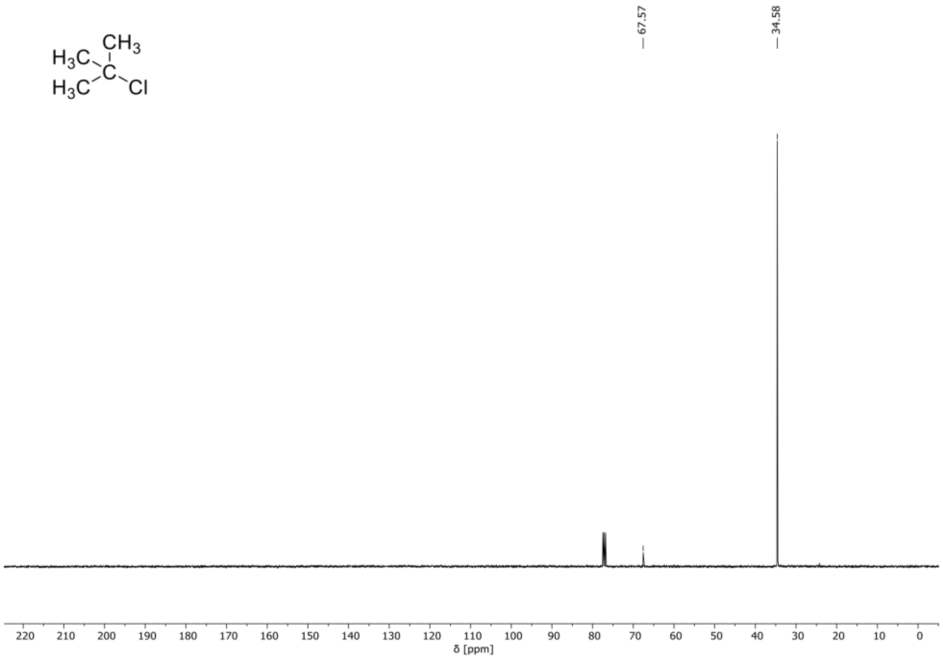

Fig. S11. ^{13}C NMR (101 MHz, $CDCl_3$, 21 °C) spectrum of *tert*-butyl chloride (tBuCl).

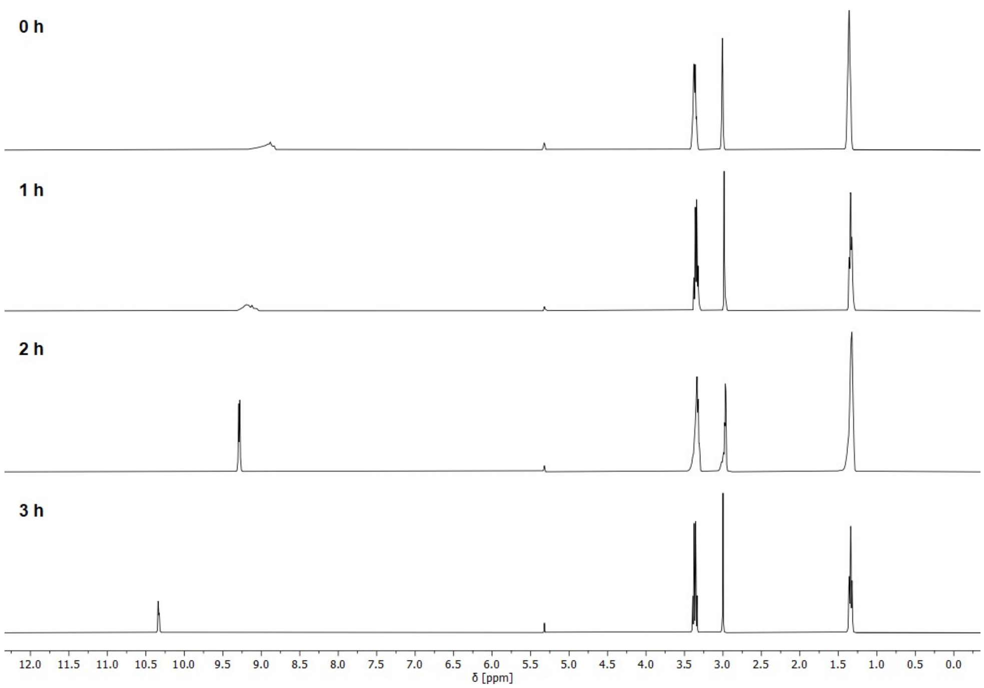

Fig. S12. ^{1}H NMR (400 MHz, $CDCl_3$, 21 °C) spectrum of the $[NEt_3Me][Cl(HCl)_n]/[NEt_3Me][Cl(Cl_2)_n]$ mixture before and during the electrolysis of $[NEt_3Me][Cl(HCl)_{2.5}]$.

Mass Spectra

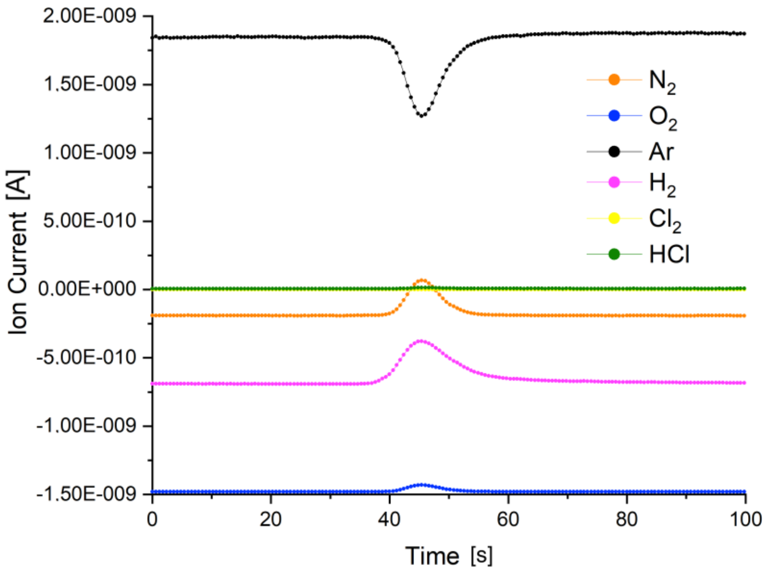

Fig. S13. Mass spectra of the gas phase after the electrolysis of $[NEt_3Me][Cl(HCl)_{2.5}]$ for 6 h.

Molecular structures in solid state

[NEt_3Me][$Cl(HCl)$]

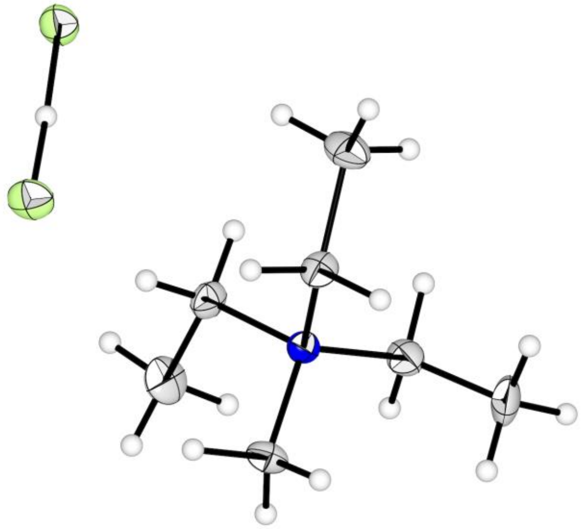

Fig. S14. Molecular structure in the solid state of [NEt_3Me][$Cl(HCl)$]. Displacement ellipsoids set at 50% probability.

[NEt_2Me_2][$Cl(HCl)$]

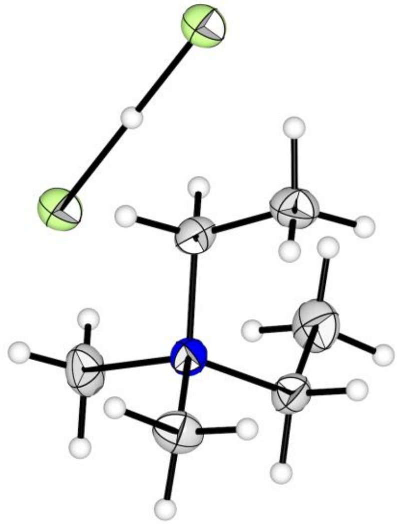

Fig. S15. Molecular structure in the solid state of [NEt_2Me_2][$Cl(HCl)$]. Displacement ellipsoids set at 50% probability.

[NPr_3Me][Cl(HCl)]

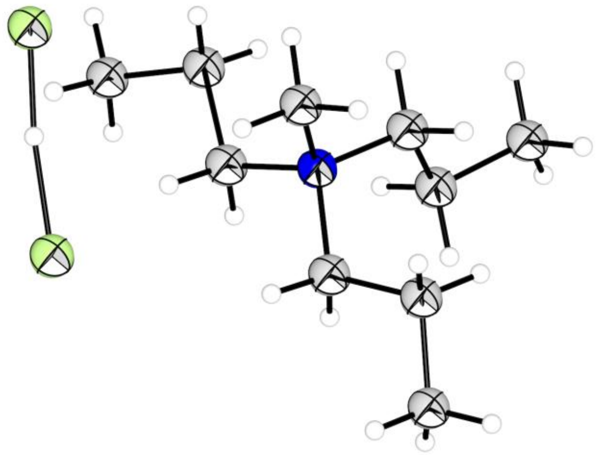

Fig. S16. Molecular structure in the solid state of [NPr_3Me][Cl(HCl)]. Displacement ellipsoids set at 50% probability.

[NBu_3Me][Cl(HCl)]

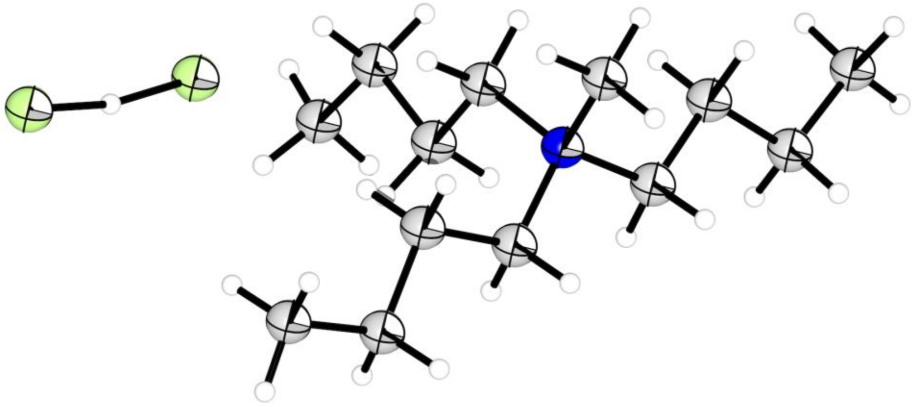

Fig. S17. Molecular structure in the solid state of [NBu_3Me][Cl(HCl)]. Displacement ellipsoids set at 50% probability.

Crystal data

Table S5. Crystal data and structure refinement for $[NEt_3Me][Cl(HCl)]$ and $[NEt_2Me_2][Cl(HCl)]$

**Empirical formula**	**$[NEt_3Me][Cl(HCl)]$**	**$[NEt_2Me_2][Cl(HCl)]$**
**Formula weight**	188.13	174.10
**T [K]**	100.00	132.00
**Crystal system**	orthorhombic	orthorhombic
**Space group**	$Pna2_1$	*Pbcm*
**a [Å]**	13.0477(7)	7.0315(5)
**b [Å]**	7.0405(3)	24.0842(17)
**c [Å]**	11.6520(6)	22.8966(16)
**α [°]**	90	90
**β [°]**	90	90
**γ [°]**	90	90
**V [Å^3]**	1070.38(9)	3877.5(5)
**Z**	4	16
**ρ_{calc} [g cm^{-3}]**	1.167	1.193
**μ [mm^{-1}]**	0.549	0.600
**F(000)**	408.0	1504.0
**Crystal size [mm^3]**	0.334 × 0.125 × 0.078	0.309 × 0.27 × 0.132
**Radiation**	MoK_α (λ = 0.71073)	MoK_α (λ = 0.71073)
**2Θ range for data collection [°]**	6.246 to 56.586	4.91 to 56.6
**Index ranges**	$-17 \leq h \leq 17$, $-9 \leq k \leq 9$, $-15 \leq l \leq 15$	$-9 \leq h \leq 9$, $-32 \leq k \leq 32$, $-30 \leq l \leq 30$
**Reflections collected**	22508	224166
**Independent reflections**	2670 [R_{int} = 0.0540, R_{sigma} = 0.0275]	4939 [R_{int} = 0.0371, R_{sigma} = 0.0101]
**Data/restraints/ parameters**	2670/12/149	4939/0/186
**Goodness-of-fit on F^2**	1.271	1.173
**Final R indexes [I>=2σ (I)]**	R_1 = 0.0445, wR_2 = 0.0869	R_1 = 0.0305, wR_2 = 0.0734
**Final R indexes [all data]**	R_1 = 0.0461, wR_2 = 0.0873	R_1 = 0.0401, wR_2 = 0.0858
**Largest diff. peak/hole [e Å^{-3}]**	0.29/–0.50	0.38/–0.33
**CCDC deposition number**	2287985	2287982

Table S6. Crystal data and structure refinement for $[NPr_3Me][Cl(HCl)]$ and $[NBu_3Me][Cl(HCl)]$

**Empirical formula**	**$[NPr_3Me][Cl(HCl)]$**	**$[NBu_3Me][Cl(HCl)]$**
**Formula weight**	230.21	272.29
**T [K]**	125.10	125.00
**Crystal system**	monoclinic	monoclinic
**Space group**	$P2_1$	$P2_1$
**a [Å]**	8.2458(4)	9.6732(5)
**b [Å]**	9.5529(5)	9.0531(6)
**c [Å]**	9.4568(5)	9.9172(7)
**α [°]**	90	90
**β [°]**	114.713(2)	106.270(2)
**γ [°]**	90	90
**V [Å^3]**	676.70(6)	833.69(9)
**Z**	2	2
**ρ_{calc} [g cm^{-3}]**	1.130	1.085
**μ [mm^{-1}]**	0.445	0.371
**F(000)**	252.0	300.0
**Crystal size [mm^3]**	0.157 × 0.106 × 0.069	0.587 × 0.511 × 0.46
**Radiation**	MoK_α (λ = 0.71073)	MoK_α (λ = 0.71073)
**2Θ range for data collection [°]**	4.742 to 56.6	4.386 to 56.6
**Index ranges**	$-10 \le h \le 10, -12 \le k \le 12, -12 \le l \le 12$	$-12 \le h \le 12, -12 \le k \le 12, -13 \le l \le 13$
**Reflections collected**	19238	29982
**Independent reflections**	3353 [R_{int} = 0.0420, R_{sigma} = 0.0272]	4137 [R_{int} = 0.0423, R_{sigma} = 0.0255]
**Data/restraints/ parameters**	3353/1/127	4137/1/154
**Goodness-of-fit on F^2**	1.136	1.076
**Final R indexes [I>=2σ (I)]**	R_1 = 0.0292, wR_2 = 0.0593	R_1 = 0.0298, wR_2 = 0.0733
**Final R indexes [all data]**	R_1 = 0.0341, wR_2 = 0.0637	R_1 = 0.0342, wR_2 = 0.0784
**Largest diff. peak/hole [e Å^{-3}]**	0.19/–0.20	0.19/–0.24
**CCDC deposition number**	2287983	2287984

Orbital diagram of 3c-4e bond

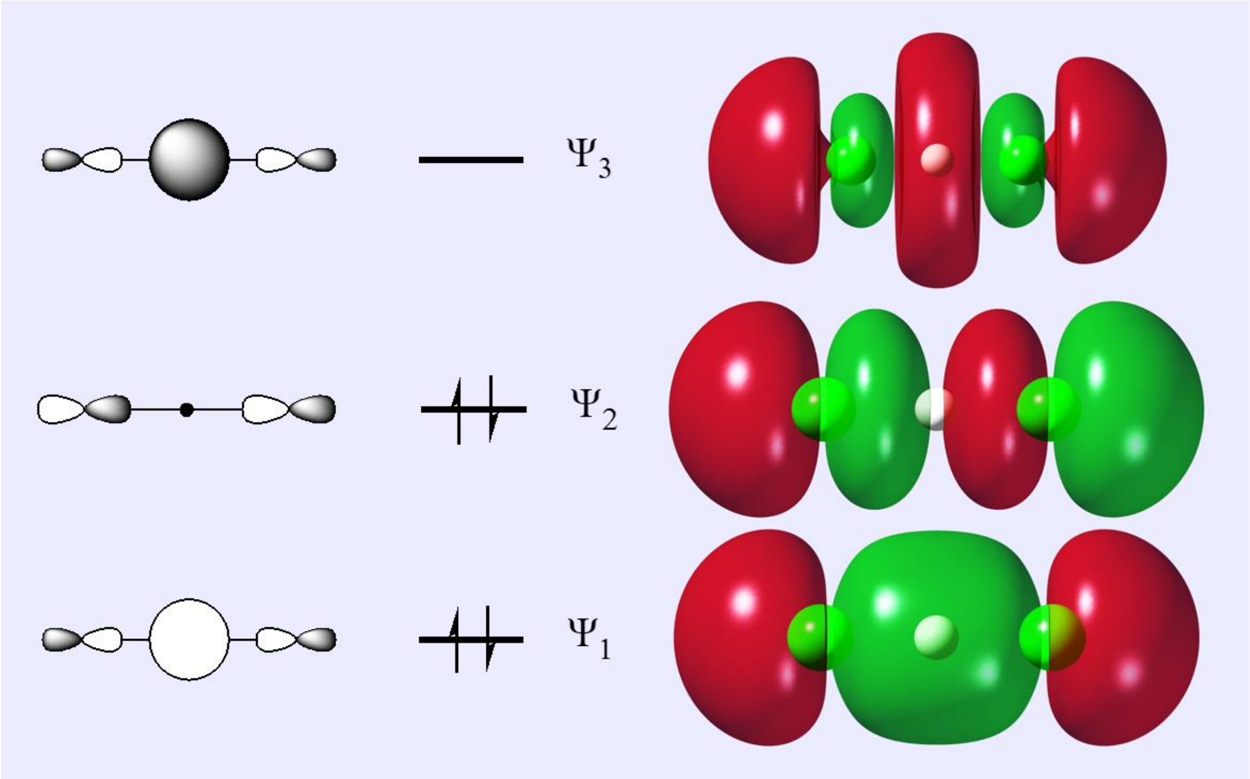

Fig. S18. Illustration of the molecular orbitals of an isolated bichloride anion $[Cl\text{-}HCl]^{-}$. The Kohn-Sham orbitals – here shown as an isosurface of 0.02 a.u. – were extracted from a single point B3LYP calculation with def2-TZVP basis set. Previously, the structure of $[Cl\text{-}HCl]^{-}$ was optimized at CCSD(T) level, yielding a Cl-H bond distance of 1.563 Å. All pictures were created with GaussView 6.0.16 based on calculations with Gaussian 16, Rev. A.03.[51]

Movie S1: Bichloride based ionic liquid

In this Movie S1 an introduction to the importance of hydrogen chloride is shown in conjunction with the handling and electrolysis of the bichloride based ionic liquid.

REFERENCES AND NOTES

1. R. Lin, A. P. Amrute, J. Pérez-Ramírez, Halogen-mediated conversion of hydrocarbons to commodities. *Chem. Rev.* **117**, 4182–4247 (2017).

2. M. Kleoff, P. Voßnacker, S. Riedel, The rise of trichlorides enabling an improved chlorine technology. *Angew. Chem. Int. Ed.* **62**, e202216586 (2023).

3. P. Schmittinger, T. Florkiewicz, L. C. Curlin, B. Lüke, R. Scannell, T. Navin, E. Zelfel, R. Bartsch, Chlorine in *Ullmanns Encyclopedia of Industrial Chemistry*, (Wiley-VCH, 2011), pp. 531–621.

4. P. Schmittinger, *Chlorine* (Wiley-VCH, 2000).

5. H. J. Arpe, *Industrielle Organische Chemie* (Wiley-VCH, 2007).

6. M. Bertau, A. Müller, P. Fröhlich, M. Katzberg, *Industrielle Anorganische Chemie* (Wiley-VCH, 2013).

7. Y. Zhao, S. Gu, K. Gong, J. Zheng, J. Wang, Y. Yan, Low-voltage gaseous HCl electrolysis with an iron redox-mediated cathode for chlorine regeneration. *Angew. Chem. Int. Ed.* **56**, 10735–10739 (2017).

8. D. R. Lide (ed.), *CRC Handbook of Chemistry and Physics, Internet Version* (CRC Press, 2005).

9. W. Bartknecht, *Explosionsschutz – Grundlagen und Anwendung* (Springer, 1993).

10. J. L. Barton, Electrification of the chemical industry. *Science* **368**, 1181–1182 (2020).

11. Z. J. Schiffer, K. Manthiram, Electrification and decarbonization of the chemical industry. *Joule* **1**, 10–14 (2017).

12. F. Klaucke, T. Karsten, F. Holtrup, E. Esche, T. Morosuk, G. Tsatsaronis, J. U. Repke, Demand response potentials for the chemical industry. *Chem. Ing. Tech.* **89**, 1133–1141 (2017).

13. C. Hoffmann, J. Hübner, F. Klaucke, N. Milojević, R. Müller, M. Neumann, J. Weigert, E. Esche, M. Hofmann, J. U. Repke, R. Schomäcker, P. Strasser, G. Tsatsaronis, Assessing the realizable flexibility potential of electrochemical processes. *Ind. Eng. Chem. Res.* **60**, 13637–13660 (2021).

14. B. Schäfer, J. Sauer, Trends der chemischen Prozessindustrie. *Chem. Ing. Tech.* **92**, 183–191 (2020).

15. M. J. Palys, P. Daoutidis, Power-to-X: A review and perspective. *Comp. Chem. Eng.* **165** 107948 (2022).

16. M. Sterner, M. Specht, Power-to-Gas and Power-to-X – the history and results of developing a new storage concept. *Energies* **14**, 6594 (2021).

17. R. Schlögl, Put the sun in the tank: Future developments in sustainable energy systems, *Angew. Chem. Int. Ed.* **58**, 343–348 (2019).

18. H. Over, R. Schomäcker, What makes a good catalyst for the Deacon process? *ACS Catal.* **3**, 1034–1046 (2013).

19. D. Teschner, G. Novell-Leruth, R. Farra, A. Knop-Gericke, R. Schlögl, L. Szentmiklósi, M. G. Hevia, H. Soerijanto, R. Schomäcker, J. Pérez-Ramírez, N. López, In situ surface coverage analysis of RuO2-catalysed HCl oxidation reveals the entropic origin of compensation in heterogeneous catalysis. *Nat. Chem.* **4**, 739–745 (2012).

20. D. Crihan, M. Knapp, S. Zweidinger, E. Lundgren, C. J. Weststrate, J. N. Andersen, A. P. Seitsonen, H. Over, Stable deacon process for HCl oxidation over RuO2. *Angew. Chem. Int. Ed.* **47**, 2131–2134 (2008).

21. K. Likit-anurak, R. White, L. A. Murdock, B. C. Benicewicz, S. Shimpalee, B. H. Meekins, Fully anhydrous HCl electrolysis using polybenzimidazole membranes. *Int. J. Hydrogen Energy* **47**, 26859–26864 (2022).

22. S. Bechtel, T. Vidaković-Koch, K. Sundmacher, Energy-efficient gas-phase electrolysis of hydrogen chloride. *Chem. Ing. Tech.* **91**, 795–808 (2019).

23. R. H. He, B. W. Long, Y. Z. Lu, H. Meng, C. X. Li, Solubility of Hydrogen Chloride in Three 1-Alkyl-3-methylimidazolium Chloride Ionic Liquids in the Pressure Range (0 to 100) kPa and Temperature Range (298.15 to 363.15) K. *J. Chem. Eng. Data* **57**, 2936–2941 (2012).

24. J. Zhu, H. Shao, L. Feng, Y. Lu, H. Meng, C. Li, Absorptive separation of HCl gas by choline chloride-based deep eutectic solvents. *J. Mol. Liq.* **341**, 116928 (2021).

25. H. Shao, J. Zhu, L. Feng, X. Liang, X. Lu, H. Meng, C. Li, Solubility behavior and the mechanism of HCl Gas in Four [EMIM]Cl-based deep eutectic solvents. *J. Chem. Eng. Data* **67**, 3097–3107 (2022).

26. T. M. Reichenau, F. Steinke, M. T. Wharmby, C. Näther, T. A. Engesser, N. Stock, Targeted synthesis of a highly stable aluminium phosphonate Metal–Organic framework showing reversible HCl ADSORPTION. *Angew. Chem. Int. Ed.* **62**, e202303561 (2023).

27. W. Xiong, Y. Lu, C. Li, J. Geng, Y. Wu, X. Hu, An imidazole-based DES serving as a “courier” for the efficient coupling of HCl capture and conversion under mild conditions. *Green Chem.* **25**, 1898–1907 (2023).

28. P. Voßnacker, N. Schwarze, T. Keilhack, M. Kleoff, S. Steinhauer, Y. Schiesser, M. Paven, S. Yogendra, R. Weber, S. Riedel, Alkyl ammonium chloride salts for efficient chlorine storage at ambient conditions. *ACS Sustainable Chem. Eng.* **10**, 9525–9531 (2022).

29. P. Voßnacker, A. Wüst, T. Keilhack, C. Müller, S. Steinhauer, H. Beckers, S. Yogendra, Y. Schiesser, R. Weber, M. Reimann, R. Müller, M. Kaupp, S. Riedel, Novel synthetic pathway for the production of phosgene. *Sci. Adv.* **7**, eabj5186 (2021).

30. P. Voßnacker, S. Steinhauer, J. Bader, S. Riedel, Synthesis and characterization of poly(hydrogen halide) halogenates (-I). *Chem. A Eur. J.* **26**, 13256–13263 (2020).

31. P. Voßnacker, S. Riedel, Storage medium for storing hydrogen chloride and method for separating and storing hydrogen chloride HCl from HCl containing gas. ***WO2023020942*** **A1** (2021).

32. G. A. Landrum, N. Goldberg, R. Hoffmann, Bonding in trihalides (X3−), mixed trihalides (X2Y−) and hydrogen bihalides (X2H−). The connection between hypervalent, electron-rich three-center, donor-acceptor and strong hydrogen bonding. *J. Chem. Soc. Dalton Trans.*, 3605–3613 (1997).

33. D. R. Stull, Vapor pressure of pure substances. organic and inorganic compounds. *Ind. Eng. Chem.* **39**, 517–550 (1947).

34. S. V. Kostjuk, Recent progress in the Lewis acid co-initiated cationic polymerization of isobutylene and 1,3-dienes. *RSC Adv.* **5**, 13125–13144 (2015).

35. P. Johnston, N. Carthey, G. J. Hutchings, Discovery, development, and commercialization of gold catalysts for acetylene hydrochlorination. *J. Am. Chem. Soc.* **137**, 14548–14557 (2015).

36. G. Malta, S. A. Kondrat, S. J. Freakley, C. J. Davies, L. Lu, S. Dawson, A. Thetfold, E. K. Gibson, D. J. Morgan, W. Jones, P. P. Wells, P. Johnston, C. R. A. Catlow, C. J. Kieley, G. J. Hutchings, Identification of single-site gold catalysis in acetylene hydrochlorination. *Science* **355**, 1399–1403 (2017).

37. S. K. Kaiser, E. Fako, I Surin, F. Krumeich, V. A. Kondratenko, E. V. Kondratenko, A. H. Clark, N. López, J. Pérez-Ramírez, Performance descriptors of nanostructured metal catalysts for acetylene hydrochlorination. *Nat. Nanotechnol.* **17**, 606–612 (2022).
38. L. Ye, X. Duan, S. Wu, T. S. Wu, Y. Zhao, A. W. Robertson, H. L. Chou, J. Zheng, T. Ayvali, S. Day, C. Tang, Y. L. Soo, Y. Yuan, S. C. E. Tsang, Self-regeneration of Au/CeO2 based catalysts with enhanced activity and ultra-stability for acetylene hydrochlorination. *Nat. Commun.* **10**, 914 (2019).
39. J. Li, H. Zhang, L. Li, M. Cai, Y. Li, D. Xie, J. Zhang, Synergistically catalytic hydrochlorination of acetylene over the highly dispersed ru active species embedded in p-containing ionic liquids. *ACS Sustainable Chem. Eng.* **8**, 10173–10184 (2020).
40. J. Zhao, Y. Yue, G. Sheng, B. Wang, H. Lai, S. Di, Y. Zhai, L. Guo, X. Li, Supported ionic liquid-palladium catalyst for the highly effective hydrochlorination of acetylene. *Chem. Eng. J.* **360**, 38–46 (2019).
41. V. Radtke, K. Pütz, D. Himmel, I. Krossing, The inverted philosopher's stone: How to turn silver to a base metal. *J. Solid State Electrochem.* **24**, 2847–2852 (2020).
42. Hydrogen Europe Position Paper on PFAS (2023).
43. X. Z. Lim, Could the world go PFAS-free? Proposal to ban 'forever chemicals' fuels debate *Nature* **620**, 24–27 (2023).
44. H. Kobler, R. Munz, G. A. Gasser, G. Simchen, Eine einfache Synthese von Tetraalkylammoniumsalzen mit funktionellen Anionen. *Justus Liebigs Ann. Chem.* **12**, 1937–1945 (1978).
45. G. M. Sheldrick, SHELXT – Integrated space-group and crystal-structure determination. *Acta Cryst. A.* **71**, 3–8 (2015).

46. G. M Sheldrick, Crystal structure refinement with SHELXL. *Acta Cryst. C* **71**, 3–8 (2015).
47. O. V. Dolomanov, L. J. Bourhis, R. J. Gildea, J. A. K. Howard, H. Puschmann, OLEX2: A complete structure solution, refinement and analysis program. *J. Appl. Cryst.* **42**, 339–341 (2009).
48. NIST Chemistry WebBook, Sadtler Research Labs Under US-EPA Contract (6 November 2023); https://webbook.nist.gov.
49. R. M. Denton, J. An, B. Adeniran, A. J. Blake, W. Lewis, A. M. Poulton, Catalytic phosphorus(V)-mediated nucleophilic substitution reactions: Development of a catalytic appel reaction. *J. Org. Chem.* **76**, 6749–6767 (2011).
50. G. Jander, K. F. Jahr, H. Knoll, Maßanalyse – Theorie und Praxis der klassischen und der elektrochemischen Titrierverfahen (Walter de Gruyter&Co, 1973).
51. M. J. Frisch, G. W. Trucks, H. B. Schlegel, G. E. Scuseria, M. A. Robb, J. R. Cheeseman, G. Scalmani, V. Barone, G. A. Petersson, H. Nakatsuji, X. Li, M. Caricato, A. V. Marenich, J. Bloino, B. G. Janesko, R. Gomperts, B. Mennucci, H. P. Hratchian, J. V. Ortiz, A. F. Izmaylov, J. L. Sonnenberg, D. Williams-Young, F. Ding, F. Lipparini, F. Egidi, J. Goings, B. Peng, A. Petrone, T. Henderson, D. Ranasinghe, V. G. Zakrzewski, J. Gao, N. Rega, G. Zheng, W. Liang, M. Hada, M. Ehara, K. Toyota, R. Fukuda, J. Hasegawa, M. Ishida, T. Nakajima, Y. Honda, O. Kitao, H. Nakai, T. Vreven, K. Throssell, J. A. Montgomery, Jr., J. E. Peralta, F. Ogliaro, M. J. Bearpark, J. J. Heyd, E. N. Brothers, K. N. Kudin, V. N. Staroverov, T. A. Keith, R. Kobayashi, J. Normand, K. Raghavachari, A. P. Rendell, J. C. Burant, S. S. Iyengar, J. Tomasi, M. Cossi, J. M. Millam, M. Klene, C. Adamo, R. Cammi, J. W. Ochterski, R. L. Martin, K. Morokuma, O. Farkas, J. B. Foresman, and D. J. Fox, *Gaussian 16, Revision A.03* (Gaussian Inc., Wallingford CT, 2016).
